# Lewis Acid–Base Synergistically Enhancing Practical Composite Electrolyte for Fluoride‐ion Batteries at Room Temperature

**DOI:** 10.1002/advs.202502824

**Published:** 2025-04-27

**Authors:** Hong Cui, Xiao Gao, Keyu Guo, Wu Liu, Bo Ouyang, Wenbin Yi

**Affiliations:** ^1^ The School of Chemistry and Chemical Engineering Nanjing University of Science and Technology Nanjing 210094 China; ^2^ The School of Science Nanjing University of Science and Technology Nanjing 210094 China

**Keywords:** fluoride‐ion batteries, quasi‐solid‐state electrolytes, room temperatures

## Abstract

Fluoride‐ion batteries (FIBs) represent a potential “next‐generation” electrochemical storage device, offering high energy density. However, the practical implementation of FIBs at room temperature is impeded by the limitations of currently available ceramic electrolytes. Here, a composite NH_4_HF_2_@PEO@β‐PbSnF_4_ electrolyte with both high conductivity of 10^−4^ S cm^−1^ and wide electrochemical stability window (4.59 V vs Pb/PbF_2_) at room temperature is fabricated. Field emission transmission electron microscope (FETEM) demonstrates the presence of a space charge region, which enhances the conductivity. Furthermore, ^19^F NMR and density functional theory (DFT) calculations elucidate that the interaction between Sn^2+^ (Lewis acid) and HF_2_^−^ (Lewis base) induces significant modifications to the electronic structure, which critically contribute to the enhanced electrochemical stability window of the composite electrolyte. Integrating this promising electrolyte with high‐voltage CuF_2_ cathodes and Pb/PbF_2_ anodes, a reversible coin cell with a discharge capacity of 143 mAh g^−1^ up to 50 cycles is demonstrated. The rational design of such composite electrolytes offers a pathway toward the practical application of FIBs at room temperature.

## Graphical abstract

A quasi‐solid‐state fluoride ion battery with various practical qualities is implemented by a salt‐polymer‐ceramic composite electrolyte. β‐PbSnF_4_, as an active filler, can greatly improve the electrochemical stability and conductivity of the electrolyte, opening up new possibilities for cutting‐edge electrolyte design for fluoride ion batteries.

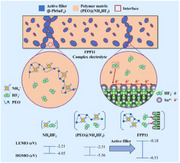

## 1 Introduction

The United States and European Union countries are proactively addressing the challenges associated with the development of lithium‐ion batteries (LIBs), including limited theoretical ceilings, expensive ingredients, and unsatisfactory safety.^[^ ^1^, ^2^, ^3^ ^]^ Through programs such as BATTERY 2030+ and The Federal Consortium for Advanced Batteries, they aim to establish practical next‐generation batteries for the future. Fluoride‐ion batteries (FIBs) are gaining traction as compelling alternatives to lithium‐ion batteries (LIBs), primarily due to their exceptionally high theoretical volumetric energy density, estimated to be roughly eightfold that of LIBs.^[^ ^4^ ^]^ However, advancing FIB technology to a commercially viable and technically superior next‐generation storage solution remains constrained by electrolyte performance. Zhang et al. indicate that the electrolyte‐side fluoride‐ion conduction is considered to be the dominant determining process at the electrode/electrolyte interface in FIBs, which means enhancing the conductivity of the electrolyte through proper design is crucial for improving the performance of FIBs toward practicality.^[^ ^5^ ^]^ Liquid electrolytes face the challenge of flammable organic solvents and the additional sealing structures further hinder the enhancement of volumetric energy density, the key advantage of FIBs. Solid electrolytes offer distinct advantages in terms of enhanced safety profiles and greater design flexibility, which are particularly beneficial for advanced energy systems.^[^ ^6^ ^]^ The intrinsic low conductivity and interfacial wettability remain a formidable barrier, as there are few ceramic solid‐state FIB electrolytes currently exhibiting practical conduction at ambient conditions.^[^ ^7^ ^]^ Addressing this constraint represents a pivotal research frontier in the development of room‐temperature‐operable FIB systems.

Inspired by the design of composite electrolytes in LIBs, ceramic fillers dispersed in the polymer matrix hinder the local reorganization of polymer chains, thereby enhancing conductivity by decreasing polymer crystallization.^[^ ^8^, ^9^ ^]^ Additionally, the use of active fillers can create a space charge region at the matrix‐filler interface, forming static pathways for conduction and decoupling conduction from polymer relaxation.^[^ ^10^, ^11^ ^]^ The space charge region at the poly(ethylene oxide) (PEO, matrix)‐Li_6.25_Ga_0.25_La_3_Zr_2_O_12_ (filler) interface was observed using a transmission electron microscope (TEM) by Li et al., verified using the phase‐field method based on the Poisson‐Cahn equations, and the electrochemical performance of the resulting composite electrolyte was significantly improved.^[^ ^12^ ^]^ This resolves the issue of low conductivity in polymers when the operating temperature is below its glass transition temperature (*T_g_*), offering a crucial solution for practical polymer‐based FIBs at room temperature (RT).

In terms of electrochemical stability, incorporating fillers can establish robust interactions with the polymer matrix through the Lewis acid‐base effect. This interaction can modify the electron transition energy levels of the matrix, specifically affecting the highest occupied molecular orbital (HOMO) and the lowest unoccupied molecular orbital (LUMO).^[^ ^13^ ^]^ As demonstrated by Wang et al., the dipole‐dipole interactions between PEO (the matrix) and Li₁.₅Al₀.₅Ge₁.₅(PO₄)₃ (the filler) can alter the electron transition energy levels of PEO, thereby increasing its oxidative decomposition potential.^[^ ^14^ ^]^ Through strategic design, the filler can also alter the electron transition energy level of the salt via the Lewis acid‐base effect, thereby delaying its decomposition.^[^ ^15^ ^]^ Sun et al. demonstrated that the numerous Lewis acid sites on the surface of sepiolite interact with PF_6_^−^ (Lewis base) to inhibit the decomposition of lithium salt anions.^[^ ^16^ ^]^ Further investigation is required to elucidate the impact of fillers on the electrochemical stability window (ESW) of composite electrolytes in FIBs.

Regarding the selection of matrix‐salt systems, Gschwind et al. explored how the chain length of polymer matrices with “C─O─C” backbones influences their electrochemical properties when doped with NH₄HF₂ salt.^[^ ^17^ ^]^ The study found that PEO with the longest chains performed poorly, as reported: “Due to the bad capacity and performance of electrolyte PEO, it was chosen to omit it from further testing.” Conversely, Savoie et al. observed that Lewis‐basic polymers like PEO facilitate slow cation and rapid anion diffusion in lithium‐ion batteries.^[^ ^18^ ^]^ Where anions are the charge carriers, the strong affinity of the polymer matrix for cations can enhance salt dissociation, increasing the carrier concentration and positioning PEO as a viable electrolyte matrix for solid‐state FIBs. Nonetheless, simultaneously achieving high conductivity (>10⁻⁴ S cm⁻¹) and a broad electrochemical stability window (>3 V) at room temperature remains a challenge for PEO‐based electrolytes.

Herein, to the best of our knowledge, we introduce the quasi‐solid‐state composite electrolyte in FIB for the first time by incorporating β‐PbSnF_4_ as an active ceramic filler (due to its high conductivity and the Lewis acid properties of Sn^2+^) into PEO@NH_4_HF_2_ (F‐PEO) matrix (HF_2_^−^ as Lewis base).^[^ ^19^, ^20^, ^21^ ^]^ With the form of space charge region and Lewis acid‐base effect, the composite quasi‐solid‐state electrolyte F‐PEO@β‐PbSnF_4_ (FPP) shows both high conductivity (10^−4^ S cm^−1^) and wide ESW (4.59 V vs Pb/PbF_2_) at RT, trace acetonitrile as the plasticizer. Based on its excellent RT properties, the CuF_2_|FPP|Pb/PbF_2_ coin cell delivers a high specific capacity of 208 mAh g^−1^ and a stable capacity of 143 mAh g^−1^ over 50 cycles.

## 2 Results and Discussion

### 2.1 Synthesis and Preparation of FPP

**Figure 1:**
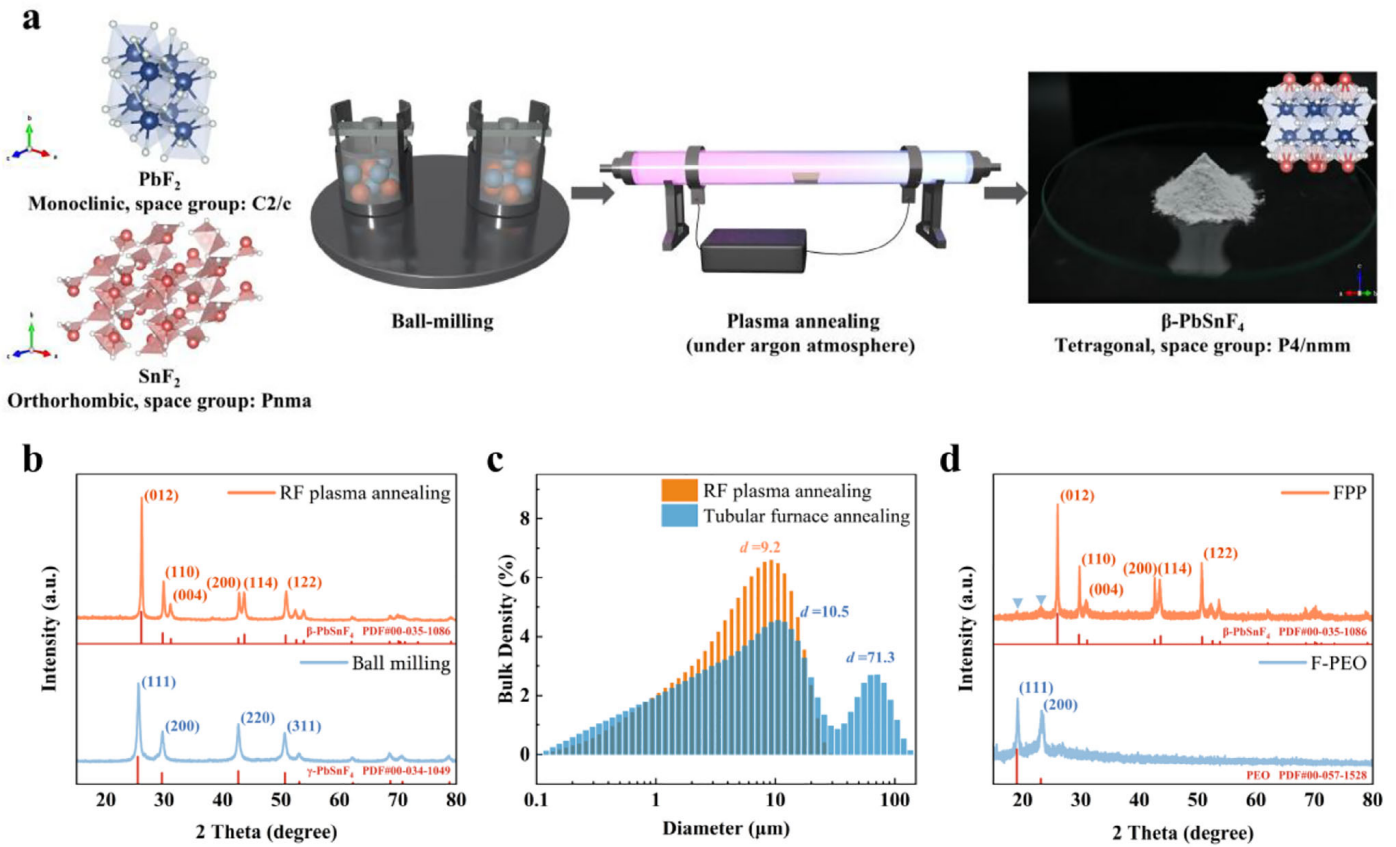
a) Synthesis process of β‐PbSnF_4_ active filler. b) XRD patterns for the products synthesized via RF plasma annealing and ball milling. c) Dry laser particle size distribution results of β‐PbSnF_4_ active filler obtained using different annealing techniques. d) XRD patterns for F‐PEO and FPP.

To obtain β‐PbSnF_4_ as an active filler, a mechanochemical method (i.e., high‐energy ball milling) is employed to synthesize the γ‐PbSnF_4_ powder, as depicted in **Figure** **1**a taking the commercial binary fluorides of PbF_2_ and SnF_2_ (with a molar ratio of 1:1) as precursors. Subsequently, the γ‐PbSnF_4_ undergoes annealing via cold plasma treatment to convert it into β‐PbSnF_4_. The X‐ray diffraction (XRD) pattern presented in Figure 1b validates the feasibility of this procedure. To the best of our knowledge, this work represents the initial endeavor to synthesize β‐PbSnF_4_ using the radio‐frequency (RF) glow discharge cold plasma technique.

As a ceramic filler dispersed in the matrix, the particle size and uniformity of the filler directly influence the performance of the composite electrolyte. A smaller particle size allows for a more adequate matrix‐filler interface to form at the same doping level, the value of this interface will be reflected later. Additionally, a narrower particle size distribution enhances the homogeneity of the composite system and ensures the reliability of the experimental results. Conventional annealing processes typically result in nucleation due to a gradual temperature rise, leading to the formation of larger grain sizes. Conversely, the spark plasma sintering (SPS) method rapidly generates high temperatures, effectively inhibiting grain growth.^[^ ^22^ ^]^ However, the high density of materials produced by SPS presents challenges in pulverization, thereby impeding the attainment of a uniform blend between the composite electrolyte polymer matrix and the ceramic filler.

The RF glow discharge cold plasma technique offers significant advantages for annealing by enabling sample preparation without the need for applied pressure.^[^ ^23^ ^]^ Meanwhile, this method facilitates electron clustering around ceramic particles, which induces repulsion and effectively prevents agglomeration, improving particle dispersion.^[^ ^24^ ^]^ The process, characterized by low temperature and rapid execution, allows ceramic materials to maintain a small grain size while minimizing inter‐particle agglomeration. This enhances the uniform dispersion of ceramic materials as active fillers within the polymer matrix. As shown in Figure 1c, laser particle size analysis indicates a notably reduced particle size distribution when utilizing plasma annealing compared to conventional methods. It is noteworthy that the results of dry laser particle size measurements (primary particles) tend to be larger than those of the actual dispersion process (secondary particles in the presence of a solvent). Thus, annealing by cold plasma does yield fillers with smaller particle sizes and narrower distributions, while the electrostatic repulsion between particles obtained by this method prevents agglomeration before mixing (single normal distribution).

As for the F‐PEO matrix, it is synthesized by adding excess NH_4_HF_2_ into PEO (Mr. 4 × 10^5^) which is dissolved in acetonitrile. The FPP composite electrolyte is formed by combining the acetonitrile‐dissolved F‐PEO matrix with the pre‐prepared β‐PbSnF_4_ active filler in a predetermined mass ratio (F‐PEO: β‐PbSnF_4_ as 2:1, 1.5:1, 1:1, 1:1.5, 2:1). Figure S1 (Supporting Information) displays the FT‐IR results of the FPP composite electrolyte alone with PEO and F‐PEO. Observed bands at 2886, 1466, 1343–1366, 1297–1240, 960, and 843 cm^−1^ in both PEO and F‐PEO correspond to the CH_2_ vibration in the PEO chain.^[^ ^25^ ^]^ Additionally, the presence of the C─O─C stretching triplet between 1200–1000 cm^−1^ is observed in all three systems. The broad peak, 3370–3110 and 720 cm^−1^ for F‐PEO should be N─H stretching vibration and out‐of‐plane bending vibration, which indicates the successful introduction of NH_4_HF_2_. Additionally, the characteristic absorption peak of the FPP composite electrolyte remains largely unchanged compared to F‐PEO, except for a new peak at 550 cm^−1^, corresponding to the β‐PbSnF_4_ powders. To further demonstrate the successful doping of β‐PbSnF_4_ active filler in the F‐PEO matrix, Figure 1d presents the XRD results of the F‐PEO matrix before and after the addition of the active filler. Prior to the addition of the filler, the F‐PEO signals were mainly attributed to PEO, withNH_4_HF_2_ almost not observed due to the formation of very small crystallites during the dispersion and recrystallization process in solution. Upon adding the filler, the diffraction peaks associated with β‐PbSnF_4_ become quite prominent, while the diffraction peaks originally attributable to F‐PEO significantly diminish. This outcome corresponds with the fact that the introduction of β‐PbSnF_4_ active filler decreases the crystallinity of the F‐PEO matrix, thereby validating the successful doping of the active filler.

**Figure 2:**
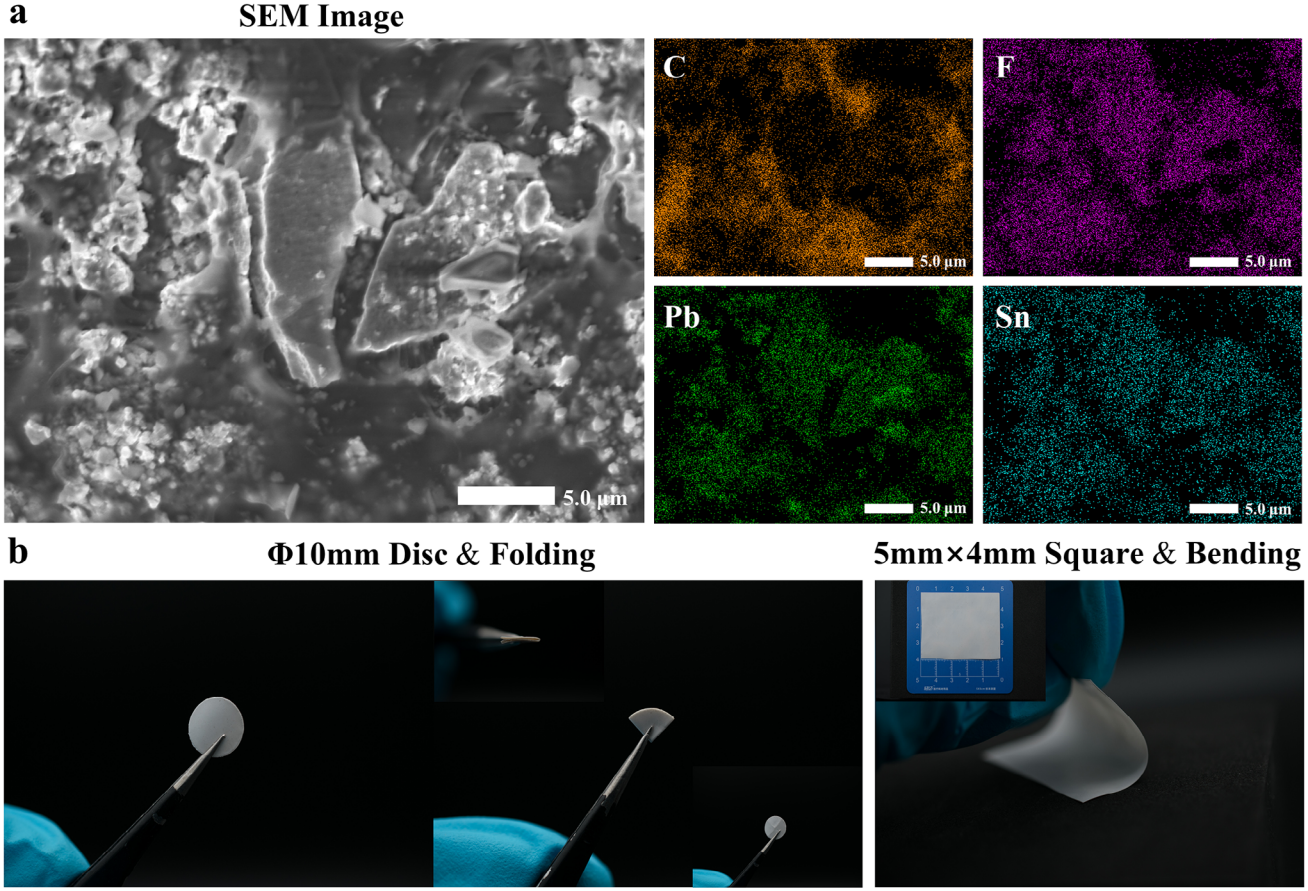
a) EDS mappings of FPP composite electrolyte. b) Images showing the bending and folding of FPP composite electrolyte film.

Ensuring the consistent distribution of the active filler throughout the polymer matrix is vital for composite electrolyte materials, forming a fundamental basis for the discussion of FPP electrolyte electrochemical properties. Figure S1 (Supporting Information) shows a scanning electron microscope (SEM) image of the FPP composite electrolyte, on which elemental mapping analysis was conducted. It demonstrates the excellent dispersion of the β‐PbSnF_4_ active filler within the PEO polymer matrix. This result further confirms that the PEO matrix is not the only component present at the electrolyte‐electrode interface in the linear scanning voltammetry (LSV) measurement, ensuring the reliability of the subsequent electrochemical stability window (ESW) results. To provide a more detailed illustration, **Figure** **2**a focuses on an enlarged view of a region that mainly contains two β‐PbSnF_4_ particles (approximate cardioid shape) ≈10 µm in size (in accordance with the laser particle size distribution result). The distribution of carbon is as expected, with fluorine uniformly distributed throughout most of the electrolyte except for significant aggregation within β‐PbSnF_4_ portions, which is reasonable for a ceramic particle. The distribution of lead and tin aligns with each other.

The results demonstrate an expected elemental distribution and uniformity in the FPP composite electrolyte, establishing a crucial foundation for additional electrochemical analyses. Figure 2b displays the photo images of the FPP electrolyte. It is evident that the FPP film can be bent and folded randomly without noticeable damage, demonstrating promising flexibility and mechanical strength. The inorganic‐organic interface between the β‐PbSnF_4_ filler and F‐PEO matrix was not observed during the bending process, suggesting excellent compatibility of the β‐PbSnF_4_ inorganic particles with the PEO host. The thickness of the FPP electrolytes used in this work is typically 180 µm, considering the universal applicability of performance characterization for composite electrolytes and the structural strength redundancy required in the experimental characterization process. Additionally, to investigate the potential benefits of FPP as a polymer‐based electrolyte in high‐specific‐energy FIBs, we discovered that a uniform electrolyte film with a thickness of 55 µm (Figure S3, Supporting Information) can be readily fabricated via casting and retains adequate structural integrity (easy to tear off from the molds). This suggests a promising approach for designing high‐specific‐energy FIB solid‐state electrolytes.

### 2.2 Electrochemical Stability of FPP

To prevent potential side reactions at the electrode‐electrolyte interface, the electrolytes need to exhibit exceptional electrochemical stability. A series of FPP composite electrolytes were prepared (F‐PEO: β‐PbSnF_4_ in ratios of 2:1, 1.5:1, 1:1, 1:1.5, 2:1) to observe the effect of active filler incorporation on the electrochemical stability of FPP composite electrolytes. LSV measurement was carried out to check the redox stability of FPP (with trace acetonitrile as plasticizer) based on an architecture of GC|FPP|Pb‐PbF_2_|Au|GC in a Swagelok cell (Figure S4, Supporting Information). The oxidation resistance test and reduction resistance test were separately conducted for each proportion of FFP composite electrolyte to reduce the influence of decomposition products during the test.

**Figure 3:**
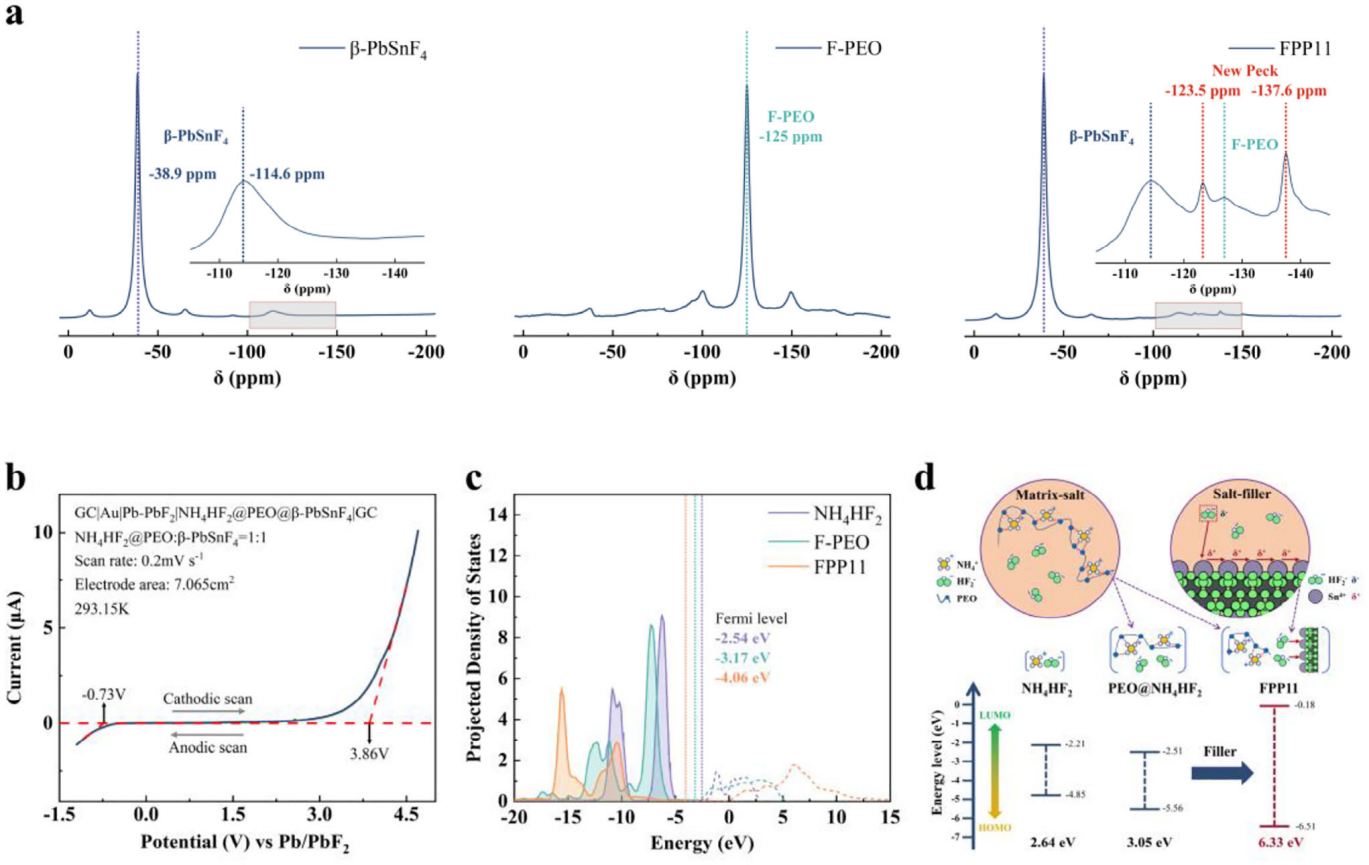
a) ^19^F MAS‐NMR results of β‐PbSnF_4_, F‐PEO and FPP11. b) Electrochemical stability (vs Pb/PbF_2_) of FPP composite electrolytes with a mass ratio of 1:1. c) PDOS results of NH_4_HF_2_, F‐PEO and FPP11. d) Visualization of interactions in NH_4_HF_2_, F‐PEO and FPP11.

The results of the LSV test are shown in Figure 3b and Figure S5 (Supporting Information). The corresponding results are summarized in Table S1 (Supporting Information), indicating that the FPP composite electrolyte with a mass ratio of F‐PEO polymer matrix to β‐PbSnF_4_ active filler of 1:1 (FPP11) has the widest ESW of 4.59 V (from ‐0.73 V to 3.86 V versus Pb/PbF_2_). Such a wide ESW is considerably larger than PEO (<3.12 V vs Pb/PbF_2_, Figure S6a, Supporting Information), NH_4_HF_2_ (<1.49 V vs Pb/PbF_2_, Figure S6b, Supporting Information), and β‐PbSnF_4_ (<0.69 V vs Pb/PbF_2_).^[^ ^26^ ^]^

The impressive wide ESW of FPP composite electrolyte, suggests a significant modification in the local chemical environment or ionic interactions within the material. To further elucidate the molecular‐level origins of this behavior, ^19^F magic‐angle spinning NMR spectroscopy was employed to investigate the chemical environment and dynamic interactions of fluorine‐containing species.

**Figure** **3**a presents the spectra of the filler and the F‐PEO before and after filler addition, along with a magnified view (inset). Isotropic signals are marked with dashed lines, while other signals correspond to spinning sidebands. The primary signals of β‐PbSnF_4_ are observed at chemical shifts of −38.9 ppm and −114.6 ppm (blue dashed lines), which are consistent with previously reported values.^[^ ^27^ ^]^ The HF_2_^−^in F‐PEO generates a signal at a chemical shift of −125 ppm (green dashed line), and the attenuation of the F‐PEO signal in FPP11 further supports the uniformity of the doping process. Notably, the FPP electrolyte formed by incorporating β‐PbSnF_4_ filler into the F‐PEO matrix exhibits two new signal peaks (red dashed lines). The new peak at a chemical shift of −123.5 ppm is attributed to the HF₂⁻ species interacting with Sn^2+^ in the filler (as Sn^2+^ exerts a stronger electron‐withdrawing effect than NH₄⁺, leading to deshielding). Meanwhile, the new peak at −137.6 ppm is of particular interest. As an anion, the high‐field shift of the HF₂⁻ signal indicates an intriguing increase in the electron density around the fluorine nuclei. This phenomenon is hypothesized to arise from the dual influence of Lewis acid sites, with HF₂⁻ (acting as a Lewis base) initially influenced by NH_4_^+^ (a Lewis acid) in the F‐PEO matrix. Upon the introduction of β‐PbSnF_4_ (with Sn^2+^ serving as an additional Lewis acid), HF₂⁻ becomes simultaneously affected by two Lewis acid centers, leading to a homogenization of the surrounding electron cloud and resulting in a shielding effect. This observation follows a similar logic to the related study of the single fluorine substituent by William R. Dolbier Jr., which documented typical shielding effects due to altered electron‐withdrawing characteristics of substituents on either side of the fluorine atom.^[^ ^28^ ^]^ Such complexity may arise from multiple interaction modes or varying local electronic environments influenced by the dual Lewis acid sites. This salt‐mediated filler‐salt‐matrix complex interaction, if present, is able to provide additional conduction pathways by forming static pathways at the filler‐matrix interface and enhancing the electrochemical stability of FPP11.^[^ ^10^ ^]^

To further explore and provide a plausible explanation for these observations, density functional theory (DFT) calculations were performed to investigate the electronic interactions and structural changes associated with HF_2_^−^ adsorption in the presence of both NH_4_^+^ and Sn^2^^+^. We performed projected density of states calculation (PDOS) on the DFT level with quantum ESPRESSO code.^[^ ^29^ ^]^ Perdew, Burke, and Ernzerhof exchange‐correlation functional and the norm‐conserving SG15 pseudopotential are used in all of our calculations with the ESPRESSO code.^[^ ^30^, ^31^, ^32^ ^]^ The cut‐off energy of wave function and charge density is chosen to be 70 Ryd and 280 Ryd respectively to guarantee convergence of electronic structure. As NH_4_HF_2_ combined with PEO can have a variety of possible structures, we used an experimental XRD‐verified LiBF_4_‐PEO structure as a starting point, replacing the Li atom with NH_4_ and the BF_4_ with HF_2_.^[^ ^33^ ^]^ All structures were relaxed with a force of <0.0005 Rydberg/Bohr. We used 3 × 3 × 1 k‐mesh for surface structure calculations and gamma point only for molecular structure calculations. As the experiments are performed in an argon gas environment, we simplified our calculations within a vacuum environment. The vacuum layer of the structure is set to be no <15Å to prevent self‐interaction between neighbor unit cells in the *z*‐direction. All the projected density of states for each structure respectively after structure relaxation.

PDOS results are shown in Figure 3c. The PDOS for NH_4_HF_2_, F‐PEO, and FPP11 are shown in purple, green, and orange lines respectively. The vacuum layer is set to zero in all our plots. The vertical dotted line indicates the Fermi level of each structure. The structural models used in the calculations are provided in Figure S7 (Supporting Information). The solid line and dashed line indicate the occupied states and unoccupied states for each structure. The occupied states are also filled with color respectively. The graph illustrates the projected density of states (PDOS) for three different materials: NH_4_HF_2_, F‐PEO, and FPP11, with the horizontal axis representing energy (in electron volts, eV) and the vertical axis representing the projected density of states.

The onset of the occupied states and unoccupied states for each structure is regarded as the LUMO and HOMO. When comparing the highest occupied molecular orbital (HOMO) levels, FPP11 exhibits the lowest HOMO level (−6.51 eV) compared to F‐PEO (−5.56 eV) and both materials have significantly lower HOMO levels than NH_4_HF_2_ (−4.85 eV). These findings imply that FPP11 possesses the greatest stability against oxidation, followed by F‐PEO, while NH₄HF₂ exhibits the highest propensity for electron activation and oxidation. Above the Fermi level, the lowest unoccupied state of the NH_4_NF_2_ molecule (LUMO: −2.21 eV) slightly decreased when the molecule is combined with PEO (−2.51 eV), and obviously increases when F‐PEO is absorbed on β‐PbSnF_4_ (−0.18 eV). This indicates that the NH_4_NF_2_ molecule requires more energy to gain the electron when combined with PEO and absorbed on β‐PbSnF_4_, which is directly related to a comparatively higher reduction resistance. The interatomic distance between Sn^2^⁺ and F⁻ in the HF₂⁻ ion, derived from the NH₄HF₂ complex with PEO adsorbed on β‐PbSnF_4_ structure, was optimized to 0.408 nm. This indicates a stable adsorbed configuration, highlighting the interaction between Sn^2+^ (acting as a Lewis acid) and HF_2_^−^ (acting as a Lewis base). The distinctive characteristics of FPP11, particularly its high LUMO and low HOMO levels, contribute to its wide ESW as depicted in Figure 3.^[^ ^34^ ^]^ Figure S8 (Supporting Information) also shows that FPP11 electrolyte has good thermal safety (5 °C min^−1^, slight mass loss at 200–300 °C can be attributed to the slight decomposition of NH_4_HF_2_; violent decomposition of the PEO matrix does not occur until 400 °C).^[^ ^35^ ^]^ Given the remarkable ESW, subsequent conductivity measurements have focused on this specific ratio.

### 2.3 Characterization of Ion Transport Properties

**Figure 4:**
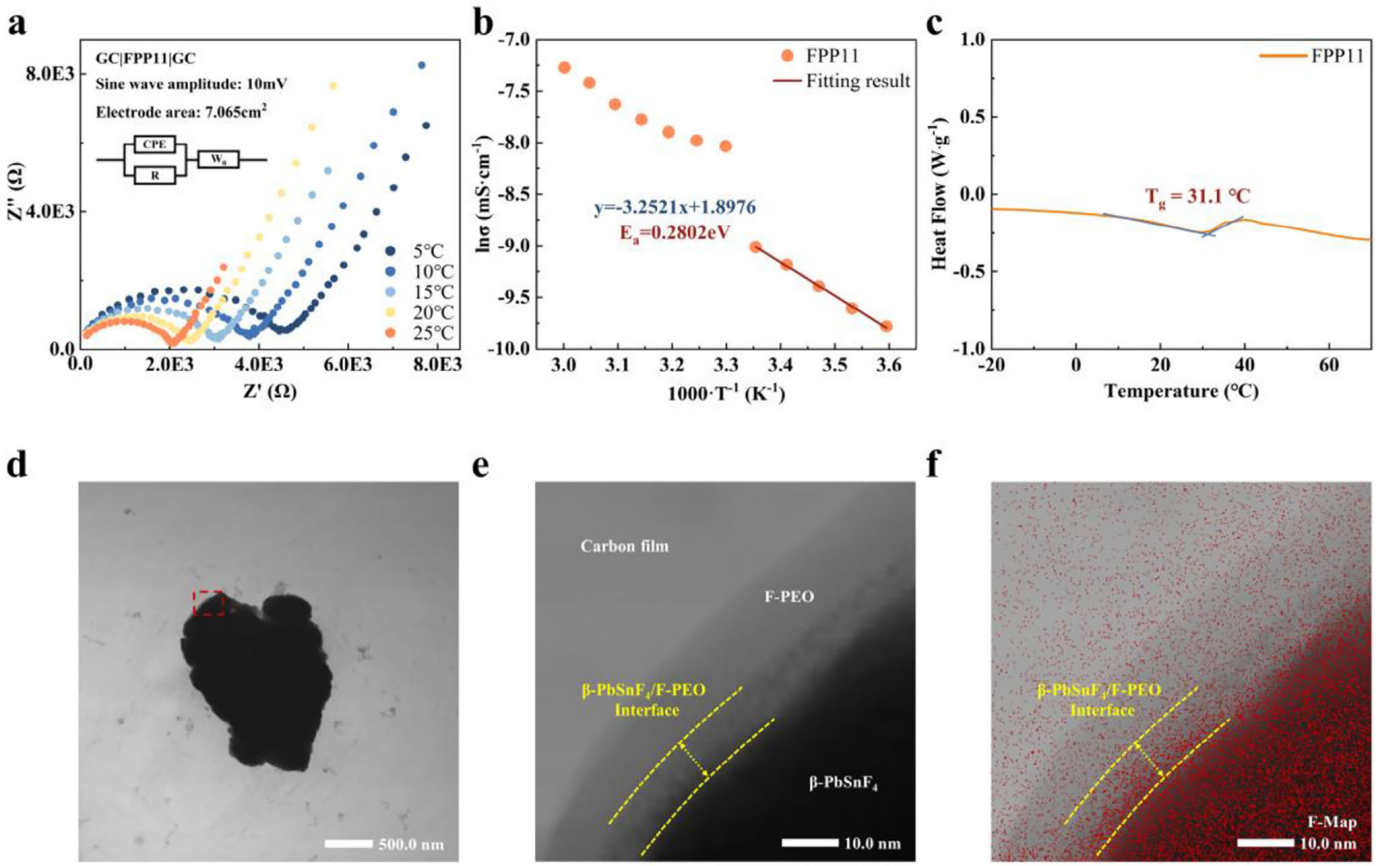
a) Nyquist plots of FPP11 composite electrolyte at different temperatures (Arrhenius behaviors). b) Arrhenius plots of conductivity and their linear fitting result of FPP11. c) DSC result of FPP11 composite electrolyte (*T_g_*). d) Field emission transmission electron microscope images of FPP11. e) Space charge region at the β‐PbSnF_4_/F‐PEO interface (close‐up view of the red‐framed area in Figure 4d). f) EDS fluorine mapping of the same region as Figure 4e.

The conductivity of FPP11 composite electrolyte is estimated based on Nyquist plots (**Figure** **4**a and Figure S9, Supporting Information) at different temperatures. Swagelok cells are used to construct a GC|FPP11|GC system for electrochemical impedance spectroscopy (EIS) testing, with trace acetonitrile as the plasticizer. The Nyquist plots exhibit a depressed semicircle in the high‐ and middle‐frequency regions and a tilted diffusion tail in the low‐frequency region. The associated equivalent circuit consists of a constant phase element (*CPE*) in parallel with a resistor *R* (both elements account for the depressed semicircle), connected in series with a Warburg impedance (*W*_0_). The Warburg impedance is expressed as diffusion tail and characterizes the polarization contribution at the electrode‐electrolyte interface (inset of Figure 4a). The conductivity at various temperatures is determined using the corresponding semicircle resistance values, which are compiled in Figure S10 (Supporting Information). To ensure accuracy, each temperature gradient was tested three times and showed significant consistency (error bar in Figure S10, Supporting Information). At 20 °C, FPP11 exhibits a conductivity of ≈10^−4^ S cm^−1^, suggesting its potential as an electrolyte for room temperature cycling in advanced FIBs.

The Arrhenius and Vogel—Tammann–Fulcher (VTF) equations are commonly used to analyze conduction in polymer‐based electrolytes.^[^ ^36^ ^]^ For low‐melting polymers like PEO, plots of *σ* against 1/*T* commonly display nonlinearity, this is due to the fact that near glass‐transition temperature (*T_g_*), the polymer matrix undergoes a transition from the glassy state to the rubbery state. In this regime, conduction mechanisms must account for polymeric chain relaxation, breathing, and segmental motion in addition to ionic hopping processes. This behavior can be modeled by combining the Arrhenius and VTF equations.^[^ ^37^ ^]^

Below the glass‐transition temperature (*T_g_*), the relationship between conductivity and temperature is commonly described using the Arrhenius model, as expressed by Equation (1):^[^ ^38^ ^]^

(1) $$\sigma =\sigma_{0}\exp\left(\frac{-E_{a}}{k_{B}T}\right)$$

(2) $$\ln\sigma =-\frac{E_{a}}{k_{B}}\frac{1}{T}+\ln\sigma_{0}$$

where *σ*_0_ represents a pre‐exponential factor, *E*_a_ is the activation energy for conductivity and *k*_B_ is the Boltzmann constant.

According to the Arrhenius equation, the ln*σ* versus 1/*T* plot displays a slope that can be used to calculate *E*_a_ by using a modified form of Equation (1), as expressed by Equation (2). The activation energy (*E*_a_) of the FPP11 composite electrolyte is determined to be 0.2802 eV from the Arrhenius plots of conductivity values (Figure 4b), which is lower than that of F‐PEO (0.2897 eV) (Figure S11, Supporting Information) and β‐PbSnF_4_ (≈0.3109 eV) within the same temperature range of 5 to 25 °C.^[^ ^39^ ^]^ Materials demonstrating linear Arrhenius variation indicate that conduction occurs via a simple hopping mechanism, independent of polymer chain breathing.^[^ ^40^ ^]^

This work primarily investigates the conductivity properties of FPP11 at RT. Additionally, to provide comprehensive insights, we briefly analyze its behavior at elevated temperatures. Above the glass‐transition temperature (*T*_g_), ionic motion becomes intertwined with structural relaxations. The VTF equation encapsulates this phenomenon by correlating conductivity with the temperature deviation from the ideal glass transition point. The VTF behavior is mathematically described by Equation (3):^[^ ^36^ ^]^

(3) $$\sigma =\sigma_{0}T^{-1/2}\;\exp\left(\frac{B}{T-T_{0}}\right)$$

where *B* represents the pseudo‐activation energy for conductivity in units of *E*_a_ */k*, and *T*_0_ denotes the reference temperature, which is typically 10 to 50 K lower than the experimental *T*_g_.

This empirical rule aligns with the differential scanning calorimetry (DSC) findings of the FPP11 composite electrolyte with trace acetonitrile as a plasticizer (Figure 4c). The significant conductivity of FPP11 composite electrolytes results from the existence of a space charge region at the filler‐matrix interface. These static pathways for conduction enable carrier migration in polymer‐based electrolytes to be separated from the movements of polymer chain segments, facilitating excellent transport performance of FPP11 composite electrolyte even at temperatures as low as 20 °C (below *T*_g_).

The importance of space charge regions in conduction was initially highlighted by Liang et al.^[^ ^41^ ^]^ They conducted a comprehensive analysis of the electrical characteristics of the two‐phase system LiI‐Al_2_O_3_, revealing significantly enhanced conduction compared to individual phases. After the discovery, strategies for optimizing the conductivity of polymer‐based electrolytes through space charge effects have garnered considerable attention.^[^ ^42^, ^43^, ^44^ ^]^ Li et al. further explored the static space charge region within the Ga‐LLZO@PEO composite electrolyte system (employing the same polymer matrix as in this work) and discussed both the generation of the space charge region and the impact of gain on conduction by combining simulations with TEM results.^[^ ^12^ ^]^ Recent studies by Gu et al. have further demonstrated that these space charge regions can significantly enhance ion transport efficiency.^[^ ^45^ ^]^

The presence of the space charge region is evidenced by the field emission transmission electron microscope (FETEM) observation in Figure 4e, similar phenomenon was observed in the findings of Li et al. as well.^[^ ^12^ ^]^ The elemental mapping result (Figure 4f) indicates a notable enrichment of fluorine in the interfacial layer (the slightly higher backing is attributed to the complete coverage of the material liquid during sample preparation). Additionally, the secondary electron image (Figure S12, Supporting Information), which excels at detecting the morphology of the sample surface, reveals that the interfacial shadow is not produced by overlapping images. This outcome illustrates the existence of a space charge region situated at the filler‐matrix interface within the FPP11 composite electrolyte, elucidating its significantly higher conductivity compared to the PEO matrix (10^−8^ to 10^−7^ S cm^−1^) at RT.^[^ ^46^ ^]^

### 2.4 Fabrication and Performance of Coin Cell

**Figure 5:**
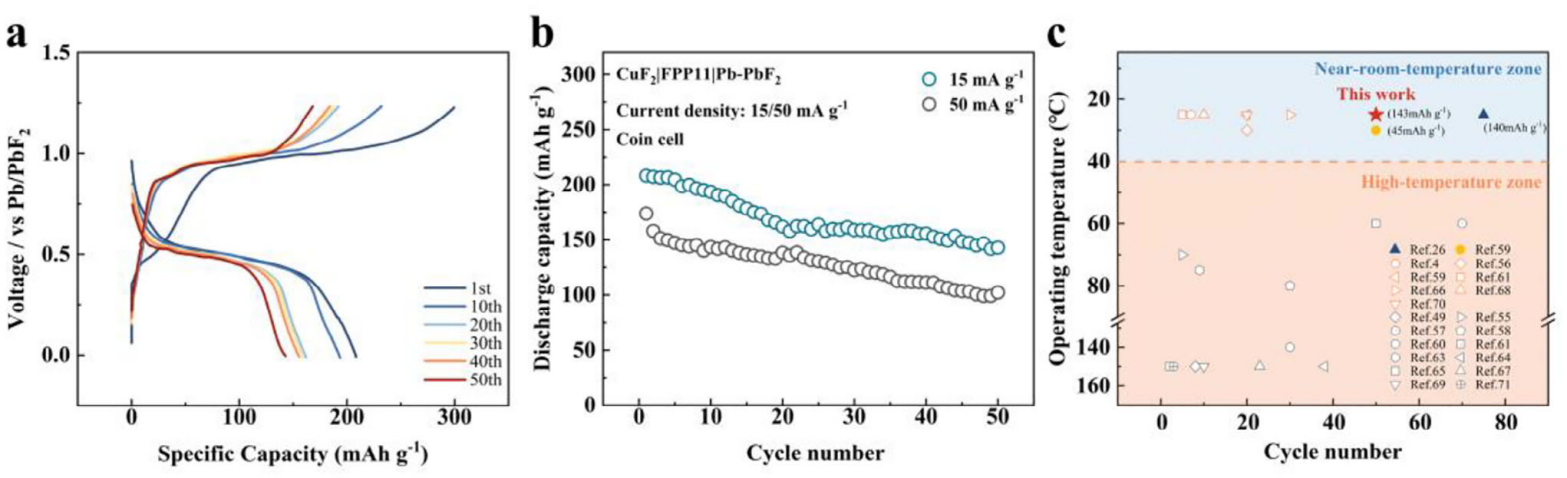
a) Charge and discharge curves (15 mA g^−1^) of Cathode|FPP11|Anode coin cell at RT. b) Cycling performances and coulombic efficiency (15 and 50 mA g^−1^)of Cathode|FPP11|Anode coin cell. c) Comprehensive cycling performance comparison for various ceramic‐based FIBs.

A coin‐cell‐type quasi‐solid‐state FIB of structure Cathode|FPP11|Anode was constructed (with trace acetonitrile as plasticizer). Here, the cathode material includes CuF_2_: PVDF: VGCF‐H (vapor‐grown carbon fiber) with a mass ratio of 4.3:2.1:3.6), while the anode includes Pb: PbF_2_: PVDF: VGCF‐H with a mass ratio of 3.5:3.5:1.2:1.8. With a high reduction potential fluoride (CuF_2_) as cathode and metallic lead as the anode (to enhance interface compatibility with the Pb(II)‐based fluoride electrolyte).^[^ ^47^ ^]^ PbF_2_ powder is also incorporated into the Pb anode to establish more reactive interfaces.^[^ ^48^ ^]^ This cell was assessed by the constant current discharge‐charge measurement at RT, under a current density of 15 mA g^−1^ (based on the mass of CuF_2_), a discharge cut‐off voltage of 0 V (vs Pb/PbF_2_), and a charge cut‐off voltage of 1.23 V versus Pb/PbF_2_. **Figure** **5**a presents the charge–discharge profiles up to 50 cycles. The initial discharge capacity starting from a voltage plateau of ≈1.05 V (vs Pb/PbF_2_) can reach 208 mAh g^−1^. Subsequent cycles show a gradual shift and decrease in voltage plateau, indicating capacity fading and increasing internal resistance over time. The 20th, 30th, 40th, and 50th cycles follow a similar trend, with each subsequent cycle demonstrating further reduced voltage plateaus and specific capacities and finally retaining 143 mAh g^−1^ of its capacity after 50 cycles (Figure 5b). The Cathode|FPP11|Anode coin cell demonstrates remarkable performance at a high‐rate capability (50 mA g⁻¹), achieving an initial discharge capacity of 173.9 mAh g⁻¹ and retaining 102.2 mAh g⁻¹ after 50 cycles, retention rate of 58.8% (Figure S13, Supporting Information). Capacity fading in high‐rate capability systems is predominantly attributed to the depletion of active materials, often caused by issues such as cracking and volumetric expansion of the Cu/CuF_2_ at elevated current rates. Overall, the graph indicates that while the cell exhibits promising specific capacity initially, there is a noticeable decline in performance over repeated cycles, highlighting areas for potential improvement in battery stability and longevity. On the one hand, although FPP11 has excellent electrochemical stability, its conductivity has just received the entry ticket for RT operation (10⁻⁴ S cm⁻¹), which requires optimization to improve conduction between the cathode and anode during cycling.^[^ ^49^ ^]^ On the other hand, there is a gap between the current capacity and the theoretical capacity of the CuF_2_ cathode. This may be attributed to the absence of a continuous conductive pathway for fluoride penetration or leaching in the cathode, leading to incomplete conversion.^[^ ^50^ ^]^ To address this, removing electrochemically inactive additives could significantly enhance the energy density (E_m_) while mitigating interfacial side reactions between heterogeneous components, extending the battery's cycle life.^[^ ^51^ ^]^ Additionally, several researches have revealed that Cu/CuF_2_ cathodes experience extreme volume expansion (up to 370%) during charge‐discharge cycles.^[^ ^52^, ^53^, ^54^ ^]^ Such volume expansion causes disorder or even cracks in cathode materials which directly contribute to irreversible capacity.

Given the importance of demonstrating both high cycling performance and operation at near room temperature simultaneously, representative FIB ceramic electrolytes were selected, and this work was juxtaposed with them.^[^ ^55^, ^56^, ^57^, ^58^, ^59^, ^60^, ^61^, ^62^, ^63^, ^64^, ^65^, ^66^, ^67^, ^68^, ^69^, ^70^, ^71^ ^]^ Figure 5c is segmented into two zones: near‐room temperature zone and high‐temperature zone, utilizing 40 °C as the dividing line. To provide a comprehensive comparison, the stable capacity of works, which is capable of >40 cycles in the near‐room‐temperature zone, is also indicated. Moreover, Table S2 (Supporting Information) compares the conductivity and the electrochemical stability of this work with those of representative ceramic electrolytes in the near‐room‐temperature zone, offering a comprehensive summary. It should be noted that there have been limited studies conducted on FIB polymer‐based electrolytes prior to this work due to inadequate conductivity and electrochemical stability, rendering them unsuitable for practical applications. Not to mention the challenge of maintaining FIB batteries over dozens of charge‐discharge cycles at RT.^[^ ^55^ ^]^ Notably, Yu et al. reported a PVA‐borax‐glycerol polymer‐based electrolyte with an excellent ionic conductivity at 30 °C (2.82 × 10⁻⁴ S cm⁻¹), comparable to that of this work (3.32×10⁻⁴ S cm⁻¹ at 30 °C).^[^ ^49^ ^]^ However, the full cell test (multiplicity transformed current densities during the test, 25–50—75—125–25 mA g^−1^) conducted by Yu et al. was performed at 60 °C (60 cycles, final capacity 69.5 mAh g^−1^) differs from the practical room‐temperature‐oriented electrolyte design of this work. Despite being among the more extensively researched FIB ceramic electrolytes, instances of operating near RT are relatively scarce. One significant work by Wang et al. (PK10) successfully utilized Ag as an anode, achieving an impressive performance of 75 cycles at 25 °C.^[^ ^26^ ^]^ However, the utilization of Ag electrodes poses a limitation for the widespread application of FIBs due to cost considerations. To avoid this, SHIMODA et al. (La_0.95_Sr_0.05_F_2.95_) also used the CuF_2_‐Pb/PbF_2_ system as this works, but can only operate at a minimum temperature of 140 °C.^[^ ^60^ ^]^ Therefore, the combination of the FPP11 electrolyte with the CuF_2_‐Pb/PbF_2_ electrode system proposed in this work exhibits competitive and practical performance compared to existing literature. This work also confirmed SHIMODA et al.’s prediction that nanoparticle CuF_2_ electrodes did have the potential for high practical capacity and stable cycling at (near) RT by using cutting‐edge solid electrolytes.^[^ ^60^ ^]^

## 3 Conclusion

In this work, a novel PEO‐based composite electrolyte, FPP11, has been successfully developed, optimized, and characterized for its electrochemical properties, demonstrating potential applications in FIBs. This composite electrolyte exhibits enhanced conductivity (1.049 × 10^−4^ S cm^−1^) at 20 °C, highlighting the foundational role of Lewis acid‐base interactions, and a significantly wide electrochemical window (4.59 V vs Pb/PbF_2_) compared to the current PbSnF_4_. The ^19^F NMR analysis showed both high‐field and low‐field shifts near the HF_2_^−^ region, indicating a complex chemical environment due to interactions between HF_2_^−^ and Sn^2+^ within the PbSnF_4_. DFT calculations confirmed that NH_4_HF_2_ adsorption on the PEO and PbSnF_4_ system significantly elevates the LUMO and lowers the HOMO, widening the electrochemical stability window (ESW). These results highlight the critical role of polymer‐salt‐filler interactions in improving electrochemical performance. The presence of such an interaction layer was also confirmed as a space charge region by FETEM, ensuring a rapid fluoride ion pathway below the polymer matrix's glass transition temperature (*T_g_*).

It is noteworthy that the CuF_2_|FPP11|Pb/PbF_2_ cell fulfills the practical requirements of RT operation, being additional‐pressure‐free (in contrast to the high pressure required for bulk‐type cells), and uses non‐precious metal electrodes, achieving a reversible capacity of 143 mAh g⁻¹ for at least 50 cycles at RT.

Based on these findings, it can be concluded that using a Lewis acid as a filler/electrolyte and a Lewis base as an electrolyte/filler, which can also provide a static ionic transport pathway while simultaneously plasticizing the matrix backbone, presents a promising approach for significantly enhancing the performance characteristics of fluoride shuttle batteries. These advancements not only improve the intrinsic properties of the composite electrolyte but also suggest new avenues for enhancing the stability and efficiency of fluoride‐ion batteries, driving forward the practical applications of these systems in energy storage technologies. Although the competitive reactions between F^−^ and HF_2_^−^ with the cathode during the reaction process remain unclear, the rational design of this cathode composite has proven valuable in achieving optimal electrochemical properties.

## 4 Experimental Section

### Materials

Poly (ethylene oxide) *M*_v_ 400 000 (372773), lead (≥99%, 209708), copper (99%, 207780), and copper (II) fluoride (98%, 217905), were purchased from Sigma‐Aldrich. Lead difluoride (AR, 99.5%, M26390) was purchased from MERYER. Acetonitrile (Water≤50 ppm, 99.9%, Safe Dry, with molecular sieves, Safe seal, 80988S) was purchased from Adamas. Ammonium hydrogen difluoride (extra pure, >95%, 208820010) and tin (II) fluoride (97.5%, 011544) were purchased from Thermo Scientific. Au foil (200 µm), coin cell case (CR2032), spacer (15.8 × 1.5 mm), and funnel spring (15.4 × 1.1 mm) were provided by Canrd. Vapor‐grown carbon fiber was purchased from Beijing CN‐FT JOVI Technology & Trading Co., Ltd. PVDF (5130, 06005501) was purchased from SHENZHEN KEJING STAR TECHNOLOGY CO., Ltd.

Unless specified otherwise, all procedures above were conducted within a high‐purity argon atmosphere glove box (MBRAUN Lab‐star Pro SP).

### Fabrication of the γ‐PbSnF_4_ Powder

To synthesize γ‐PbSnF_4_ powder, α‐PbF_2_, and SnF_2_ were blended in a 1:1 molar ratio and placed in a sealed ball milling jar, adding zirconia balls (8 mm in diameter) with a ball‐to‐material mass ratio of 20:1. Subsequently, the jar was removed from the glove box, secured tightly, and subjected to ball milling in a planetary ball mill operating at 370 rpm for 6 h at RT to achieve full synthesis of γ‐PbSnF_4_.

### Fabrication of the β‐PbSnF_4_ Powder

Afterward, γ‐PbSnF_4_ powder will be transformed to β‐PbSnF_4_ by cold plasma annealing. Both RF power supply (VERG‐2000) and RF auto match (VENA‐2000) are purchased from K‐mate Electronics LTD. With a quartz tube serving as the plasma container and positioned within a Faraday cage, photographs depicting the section in both standby and operating states are presented in Figures S14, S15 (Supporting Information). At a power of 400 W under an argon atmosphere (30 Pa), the relationship between plasma temperature and the distance from the anode was investigated using an infrared thermometer, and the results are shown in Figure S16 (Supporting Information). Consistent with the reported transformation process at 150 °C, γ‐PbSnF_4_ powder was uniformly dispersed flat in a porcelain boat measuring 4 cm in length, positioned 6–10 cm away from the anode, and treated for 10 min under the above conditions.^[^ ^72^ ^]^

### Fabrication of the FPP Electrolyte

To produce the composite FPP electrolyte, the F‐PEO matrix was first prepared by adding an excess of NH_4_HF_2_ salt to PEO, using acetonitrile as the solvent. The mixture was placed in a sealed polyethylene jar, heated to 60 °C, and stirred at 300 rpm for up to 6 h to achieve a transparent and homogeneous solution. Subsequently, the solution was vacuum‐dried for 24 h to obtain a uniform F‐PEO matrix. Next, F‐PEO and β‐PbSnF_4_ were individually weighed according to the predetermined mass ratio (2:1, 1.5:1, 1:1, 1:1.5, 2:1) and dissolved in a sealed polyethylene jar with acetonitrile. The mixture was then stirred at 300 rpm for up to 6 h at 60 °C to ensure uniform dispersion of the filler. It is crucial to avoid excessive solvent as it may cause the filler to settle slowly during subsequent drying. Through the experiments, it was determined that using 8 mL of acetonitrile per gram of F‐PEO matrix was optimal.

### X‐Ray Powder Diffraction

X‐ray powder diffraction (XRD, Bruker D8 ADVANCE) was employed with a range of diffraction angle 2*θ* = 15–80° at a scan rate of ≈3° min^−1^ at RT. Using copper as the target, the wavelength was ≈0.154 nm.

### Particle Size Measurement

Laser diffraction particle size (Master size 3000+ Pro) was measured using an automatic wet dispersion device (Hydro MV) with ultrapure water as the dispersing medium. The refractive index was set to 1.76 and the dispersion speed was set to 300 rpm. The samples were tested repeatedly after 10 min of dispersion until the data stabilized and the residuals were below 0.8 percent.

### Microcosmic Observation

A field emission scanning electron microscope (FE‐SEM, Gemini SEM 360) equipped with an energy dispersive spectrometer was used to characterize the samples' morphology. The extra high tension (EHT) was set to 10 kV and the working distance (WD) was 6.7 mm. It is worth noting that the samples were not sprayed with gold. And field emission transmission electron microscope (FETEM, JEM‐F200, cold field‐emission electron gun, 200 kV) was used to characterize the samples' morphology.

### Electrochemical Measurement

The electrochemical stability window (ESW) of the prepared sample was analyzed using linear sweep voltammetry (LSV, BioLogic SP‐300). The LSV measurements were performed at a sweep rate of 0.2 mV s^−1^ using a Pb/PbF_2_ pressed powder pellet (mass proportion 1:1) as both the counter and reference electrode, with gold flakes serving as the fluid collector.

Electrochemical impedance spectroscopy (EIS, BioLogic SP‐300) was measured using a glassy carbon electrode from 5 to 60 °C with the use of Swagelok cell test kits. The frequency ranges from 1 Hz to 7 MHz with a perturbation of 10 mV. It was worth noting that, to ensure the accuracy of the results at a given temperature, a pre‐experiment was conducted. Following the establishment of a specific temperature, electrochemical impedance spectroscopy (EIS) tests were performed at intervals of 5 min until the results of three successive tests showed close consistency. In the system environment used in this work, it took ≈30 min to reach equilibrium for every 5 °C increase. The equivalent circuit was constructed using the analysis software EC‐Lab, which was also utilized to extract the film resistance (*R*_f_).

### Spectroscopy

Fourier transform infrared (FT‐IR, Thermo NICOLETIS20, GTGS KBr detector) was recorded between 520 and 4000 cm^−1^. With an optical velocity of 0.4747; set the aperture as 150.0; used 4.000 resolution and sample scanned 32 times.

### Thermal Property

Glass transition temperature was measured by differential scanning calorimetry (DSC, METTLER DSC 3^+^), carried out at a temperature range of −30 to 70 °C under a nitrogen atmosphere and with a heating rate of 2.5 °C min^−1^. As for thermal stability, it was also evaluated using thermogravimetric analysis and differential scanning calorimetry (TG‐DSC, METTLER TGA/DSC 3^+^), carried out at a temperature range of 30 to 600 °C under a nitrogen atmosphere and with a heating rate of 5 °C min^−1^. To ensure the experiment's atmospheric purity, the system was pre‐aerated for 5 min prior to the commencement of the experiment.

### Solid‐State NMR

^19^F magic‐angle spinning NMR (MAS‐NMR, Bruker Advance‐III WB 400 MHz) was performed with a 4 mm MASDVT400W1 BL4 X/Y/F‐H probe. The sample was spun at 10 kHz, and the data was analyzed by Topspin 3.5 pl 7.

### Electrochemical Measurement—Coin‐cell Charge–Discharge Test

The cathode composition included CuF_2_: PVDF: VGCF‐H in a mass ratio of 4.3:2.1:3.6, while the anode composition included Pb: PbF_2_: PVDF: VGCF‐H in a mass ratio of 3.5:3.5:1.2:1.8. A homogeneous and well‐dispersed mixture of Pb and PbF_2_ was essential for achieving a stable electrode potential, minimizing overpotential during cycling, and providing a reservoir of fluoride ions. Both electrode pastes were coated onto gold foils. Subsequently, the FPP11 electrolyte and the coated electrodes were loaded into a coin cell. The charge–discharge capability of the samples was evaluated using a standard battery test instrument (HJ‐SD8, Hokuto Denko) under a current density of 15 mA g^−1^. The capacities were calculated referring to the weight of the active material in the cathode composite (CuF_2_).

## Conflict of Interest

The authors declare no conflict of interest.

## Supporting information



Supporting Information

## Data Availability

The data that support the findings of this study are available in the supplementary material of this article.

## References

[1] Z. Ning , G. Li , D. L. R. Melvin , Y. Chen , J. Bu , D. Spencer‐Jolly , J. Liu , B. Hu , X. Gao , J. Perera , C. Gong , S. D. Pu , S. Zhang , B. Liu , G. O. Hartley , A. J. Bodey , R. I. Todd , P. S. Grant , D. E. J. Armstrong , T. J. Marrow , C. W. Monroe , P. G. Bruce , Nature 2023, 618, 287. 37286650 10.1038/s41586-023-05970-4

[2] H. Wang , K. Feng , P. Wang , Y. Yang , L. Sun , F. Yang , W. Q. Chen , Y. Zhang , J. Li , Nat. Commun. 2023, 14, 1246. 36870994 10.1038/s41467-023-36957-4 PMC9985616

[3] K. Liu , Y. Liu , D. Lin , A. Pei , Y. Cui , Sci. Adv. 2018, 4, aas9820. 10.1126/sciadv.aas9820 PMC6014713 29942858

[4] V. K. Davis , C. M. Bates , K. Omichi , B. M. Savoie , N. Momcilovic , Q. M. Xu , W. J. Wolf , M. A. Webb , K. J. Billings , N. H. Chou , S. Alayoglu , R. K. McKenney , I. M. Darolles , N. G. Nair , A. Hightower , D. Rosenberg , M. Ahmed , C. J. Brooks , T. F. Miller , R. H. Grubbs , S. C. Jones , Science 2018, 362, 1144. 30523107 10.1126/science.aat7070

[5] D. Zhang , H. Nakano , K. Yamamoto , K. Tanaka , T. Yahara , K. Imai , T. Mori , H. Miki , S. Nakanishi , H. Iba , T. Watanabe , T. Uchiyama , K. Amezawa , Y. Uchimoto , ACS Appl. Mater. Interfaces 2021, 13, 30198. 34152731 10.1021/acsami.1c06947

[6] W. Liu , D. Lin , J. Sun , G. Zhou , Y. Cui , ACS Nano 2016, 10, 11407. 28024352 10.1021/acsnano.6b06797

[7] S. Shafiei Kaleibari , Q. Ye , M. Ni , Int. J. Energy Res. 2022, 46, 17848.

[8] S. Liu , W. Liu , D. Ba , Y. Zhao , Y. Ye , Y. Li , J. Liu , Adv. Mater. 2023, 35, 2110423. 10.1002/adma.202110423 35949194

[9] X. Wang , S. Huang , Y. Peng , Y. Min , Q. Xu , ChemSusChem 2024, 17, 202301262. 10.1002/cssc.202301262 38415928

[10] J. Zheng , Y. Y. Hu , ACS Appl. Mater. Interfaces 2018, 10, 4113. 29303244 10.1021/acsami.7b17301

[11] J. Zhang , N. Zhao , M. Zhang , Y. Li , P. K. Chu , X. Guo , Z. Di , X. Wang , H. Li , Nano Energy 2016, 28, 447.

[12] Z. Li , H. M. Huang , J. K. Zhu , J. F. Wu , H. Yang , L. Wei , X. Guo , ACS Appl. Mater. Interfaces 2019, 11, 784. 30525410 10.1021/acsami.8b17279

[13] Z. Zhang , J. Wang , H. Ying , S. Zhang , P. Huang , Z. Zhang , H. Xie , G. Han , W. Q. Han , Chem. Eng. J. 2023, 451, 138680.

[14] C. Wang , Y. Yang , X. Liu , H. Zhong , H. Xu , Z. Xu , H. Shao , F. Ding , ACS Appl. Mater. Interfaces 2017, 9, 13694. 28334524 10.1021/acsami.7b00336

[15] L. Su , Y. Zhu , X. Zhan , K. Yu , T. Guo , K. Gu , H. Wu , L. Wang , Y. Wang , X. Wang , J. Mater. Chem. A 2024, 12, 5768.

[16] X. Sun , J. Guo , X. Zhi , J. Xu , Y. Bian , K. Hou , X. Li , L. Wang , G. Liang , Colloids Surf., A 2024, 691, 133925.

[17] F. Gschwind , J. Bastien , J. Mater. Chem. A 2015, 3, 5628.

[18] B. M. Savoie , M. A. Webb , T. F. Miller 3rd, J. Phys. Chem. Lett. 2017, 8, 641. 28075599 10.1021/acs.jpclett.6b02662

[19] J. Liu , L. Yi , X. Chen , Y. Tang , Z. Zang , C. Zou , P. Zeng , D. Li , J. Xia , S. Ni , X. Wang , ACS Appl. Mater. Interfaces 2023, 15, 36373. 37482949 10.1021/acsami.3c07382

[20] Y. Liu , T. Li , S. Qiao , Z. Heng , T. Zhao , H. Wu , T. Xiong , J. Li , X. Yao , L. Long , Y. Xiang , Q. Liu , L. Lu , T. Liang , J. Chen , F. Jin , ACS Appl. Mater. Interfaces 2023, 15, 25604. 37192272 10.1021/acsami.3c04005

[21] M. De Rosa , D. Arnold , D. Hartline , L. Truong , R. Verner , T. W. Wang , C. Westin , J. Org. Chem. 2015, 80, 12288. 26575797 10.1021/acs.joc.5b02192

[22] R. Kruzel , T. Dembiczak , J. Wachowicz , MaterialsMaterials 2023, 16, 5539.

[23] J. Stockemer , P. Vanden Brande , P. V. Brande , Metall. Mater. Trans. A 2003, 34, 1341.

[24] A. Sonawane , M. A. Mujawar , S. Bhansali , ECS Trans. 2019, 88, 197.

[25] M. A. Morsi , G. M. Asnag , A. S. Assran , R. Alwafi , A. E. Tarabiah , N. A. Alshehri , A. N. Al‐Hakimi , A. Saeed , J. Energy Storage 2024, 88, 111554.

[26] J. Wang , J. Hao , C. Duan , X. Wang , K. Wang , C. Ma , Small 2022, 18, 2104508.

[27] M. Murakami , F. Fujisaki , Y. Morita , Solid State Ionics 2020, 355, 115398.

[28] W. R. Dolbier Jr. , in Guide to Fluorine NMR for Organic Chemists, Wiley, Hoboken 2016.

[29] P. Giannozzi , S. Baroni , N. Bonini , M. Calandra , R. Car , C. Cavazzoni , D. Ceresoli , G. L. Chiarotti , M. Cococcioni , I. Dabo , A. Dal Corso , S. de Gironcoli , S. Fabris , G. Fratesi , R. Gebauer , U. Gerstmann , C. Gougoussis , A. Kokalj , M. Lazzeri , L. Martin‐Samos , N. Marzari , F. Mauri , R. Mazzarello , S. Paolini , A. Pasquarello , L. Paulatto , C. Sbraccia , S. Scandolo , G. Sclauzero , A. P. Seitsonen , et al., J. Phys.‐Conden. Matt. 2009, 21, 395502. 10.1088/0953-8984/21/39/395502 21832390

[30] J. P. Perdew , K. Burke , M. Ernzerhof , Phys. Rev. Lett. 1996, 77, 3865. 10062328 10.1103/PhysRevLett.77.3865

[31] D. R. Hamann , Phys. Rev. B 2013, 88, 085117.

[32] M. Schlipf , F. Gygi , Comput. Phys. Commun. 2015, 196, 36.

[33] Y. G. Andreev , V. Seneviratne , M. Khan , W. A. Henderson , R. E. Frech , P. G. Bruce , Chem. Mater. 2005, 17, 767.

[34] A. S. Moraes , G. A. Pinheiro , T. C. Lourenço , M. C. Lopes , M. G. Quiles , L. G. Dias , J. L. F. Da Silva , J. Chem. Inf. Model. 2022, 62, 4702. 36122418 10.1021/acs.jcim.2c00748

[35] S. Rana , R. Kumar , R. S. Bharj , Chem. Eng. J. 2023, 463.

[36] K. M. Diederichsen , H. G. Buss , B. D. McCloskey , Macromolecules 2017, 50, 3831.

[37] Z. Li , J. Fu , X. Zhou , S. Gui , L. Wei , H. Yang , H. Li , X. Guo , Adv. Sci. (Weinh) 2023, 10, e2201718. 36698303 10.1002/advs.202201718 PMC10074084

[38] E. Quartarone , P. Mustarelli , Chem. Soc. Rev. 2011, 40, 2525. 21253642 10.1039/c0cs00081g

[39] M. M. Ahmad , K. Yamada , T. Okuda , J.f Phys.‐Condens. Matter 2002, 14, 7233.

[40] M. A. Ratner , P. Johansson , D. F. Shriver , MRS Bull. 2000, 25, 31.

[41] C. C. Liang , J. Electrochem. Soc. 1973, 120, 1289.

[42] K. Fu , Y. H. Gong , J. Q. Dai , A. Gong , X. G. Han , Y. G. Yao , C. W. Wang , Y. B. Wang , Y. N. Chen , C. Y. Yan , Y. J. Li , E. D. Wachsman , L. B. Hu , Proc. Natl. Acad. Sci. USA 2016, 113, 7094.27307440

[43] J. Bae , Y. T. Li , J. Zhang , X. Y. Zhou , F. Zhao , Y. Shi , J. B. Goodenough , G. H. Yu , Angew. Chemie‐Int. Ed. 2018, 57, 2096. 10.1002/anie.201710841 29314472

[44] X. Wang , H. Zhai , B. Qie , Q. Cheng , A. Li , J. Borovilas , B. Xu , C. Shi , T. Jin , X. Liao , Y. Li , X. He , S. Du , Y. Fu , M. Dontigny , K. Zaghib , Y. Yang , Nano Energy 2019, 60, 205.

[45] Z. Gu , J. Ma , F. Zhu , T. Liu , K. Wang , C. W. Nan , Z. Li , C. Ma , Nat. Commun. 2023, 14, 1632. 36964134 10.1038/s41467-023-37313-2 PMC10039002

[46] H. Chen , D. Adekoya , L. Hencz , J. Ma , S. Chen , C. Yan , H. J. Zhao , G. L. Cui , S. Q. Zhang , Adv. Energy Mater. 2020, 10, 2000049.

[47] A. W. Xiao , G. Galatolo , M. Pasta , Joule 2021, 5, 2823.

[48] C. Rongeat , M. A. Reddy , T. Diemant , R. J. Behm , M. Fichtner , J. Mater. Chem. A 2014, 2, 20861.

[49] H. Bhatia , D. T. Thieu , A. H. Pohl , V. S. K. Chakravadhanula , M. H. Fawey , C. Kübel , M. Fichtner , ACS Appl. Mater. Interfaces 2017, 9, 23707. 28570050 10.1021/acsami.7b04936

[50] Y. Yu , G. Li , C. Li , Adv. Funct. Mater. 2024, 35, 2410891.

[51] L. Cui , S. Zhang , J. Ju , T. Liu , Y. Zheng , J. Xu , Y. Wang , J. Li , J. Zhao , J. Ma , J. Wang , G. Xu , T. S. Chan , Y. C. Huang , S. C. Haw , J. M. Chen , Z. Hu , G. Cui , Nat. Energy 2024, 9, 1084.

[52] D. Zhang , K. Yamamoto , Z. Cao , Y. Wang , Z. Zhong , H. Kiuchi , T. Watanabe , T. Matsunaga , K. Nakanishi , H. Miki , H. Iba , Y. Harada , K. Amezawa , K. Maeda , H. Kageyama , Y. Uchimoto , J. Am. Chem. Soc. 2025, 147, 5649. 39804710 10.1021/jacs.4c12391

[53] Z. Cao , K. Yamamoto , T. Matsunaga , T. Watanabe , M. Kumar , N. Thakur , R. Ohashi , S. Tachibana , H. Miki , K. Ide , H. Iba , H. Kiuchi , Y. Harada , Y. Orikasa , Y. Uchimoto , Chem. Mater. 2024, 36, 1928.

[54] Z. Cao , K. Yamamoto , T. Matsunaga , M. Kumar , N. Thakur , T. Watanabe , K. Nakanishi , H. Miki , H. Iba , K. Amezawa , H. Kageyama , Y. Uchimoto , ACS Appl. Energy Mater. 2024, 7, 6640.

[55] L. Liu , L. Yang , M. Liu , X. Wang , X. Li , D. Shao , K. Luo , Z. Luo , G. Chen , J. Energy Storage 2019, 25, 100886.

[56] L. Liu , L. Yang , D. S. Shao , K. L. Luo , C. F. Zou , Z. G. Luo , X. Y. Wang , Ceram. Int. 2020, 46, 20521.

[57] Y. Yu , M. Lei , D. Li , C. Li , Adv. Energy Mater. 2023, 13, 2203168.

[58] H. Nakano , T. Matsunaga , T. Mori , K. Nakanishi , Y. Morita , K. Ide , K.‐i. Okazaki , Y. Orikasa , T. Minato , K. Yamamoto , Z. Ogumi , Y. Uchimoto , Chem. Mater. 2020, 33, 459.

[59] Z. Zang , J. Liu , X. Tao , C. Zou , X. Chen , L. Yi , B. Chang , X. Wang , J. Electroanal. Chem. 2023, 930, 117145.

[60] K. Shimoda , Y. Morita , K. Noi , T. Fukunaga , Z. Ogumi , T. Abe , ACS Energy Lett. 2023, 8, 2570.

[61] J. Liu , L. Yi , X. Chen , D. Li , S. Ni , J. Xia , L. Yang , X. Wang , Sustainable Mater. Technol 2024, 39, e00810.

[62] I. Mohammad , R. Witter , M. Fichtner , M. A. Reddy , ACS Appl. Energy Mater. 2019, 2, 1553.

[63] A. Inoo , J. Inamoto , Y. Matsuo , ACS Appl. Mater. Interfaces 2022, 14, 56678. 36472913 10.1021/acsami.2c13070

[64] M. A. Reddy , M. Fichtner , J. Mater. Chem. 2011, 21, 17059.

[65] C. Rongeat , M. A. Reddy , R. Witter , M. Fichtner , J. Phys. Chem. C 2013, 117, 4943.

[66] J. Liu , L. Yi , P. Zeng , C. Zou , X. Chen , X. Tao , X. Liu , L. Yang , Z. Zang , B. Chang , Y. Shen , X. Wang , Energy Fuels 2022, 36, 15258.

[67] D. T. Thieu , M. H. Fawey , H. Bhatia , T. Diemant , V. S. K. Chakravadhanula , R. J. Behm , C. Kübel , M. Fichtner , Adv. Funct. Mater. 2017, 27, 1701051.

[68] L. Liu , L. Yang , M. Liu , X. Li , D. Shao , K. Luo , X. Wang , Z. Luo , J. Alloys Compd. 2020, 819, 152983.

[69] I. Mohammad , R. Witter , M. Fichtner , M. A. Reddy , ACS Appl. Energy Mater. 2018, 1, 4766. 10.1021/acsami.8b04108 29741368

[70] Z. H. Zang , L. Liu , L. Yang , K. L. Luo , C. F. Zou , X. Y. Chen , X. Y. Tao , Z. G. Luo , B. B. Chang , X. Y. Wang , ACS Sustainable Chem. Eng. 2021, 9, 12978.

[71] A. Grenier , A. G. P. Gutierrez , H. Groult , D. Dambournet , J. Fluorine Chem. 2016, 191, 23.

[72] F. Fujisaki , K. Mori , M. Yonemura , Y. Ishikawa , T. Kamiyama , T. Otomo , E. Matsubara , T. Fukunaga , J. Solid State Chem. 2017, 253, 287.

