# Supplementary material for: Lewis Acid–Base Synergistically Enhancing Practical Composite Electrolyte for Fluoride‐ion Batteries at Room Temperature
Source: Adv Sci (Weinh). 2025 Apr 27;12(27):2502824. doi: 10.1002/advs.202502824 (PMC12279211; doi:10.1002/advs.202502824)
Supplement: Supplementary file 1 — Supporting Information [file ADVS-12-2502824-s001.docx]

Supporting Information

Lewis Acid–Base Synergistically Enhancing Practical Composite Electrolyte for Fluoride-ion Batteries at Room Temperature

Hong Cui, Xiao Gao^*^, Keyu Guo, Wu Liu, Bo Ouyang^*^, and Wenbin Yi^*^

H. Cui, X. Gao, K. Guo, W. Liu, W. Yi

The School of Chemistry and Chemical Engineering, Nanjing University of Science and Technology, Nanjing 210094, China

E-mail: gaoxiao@njust.edu.cn, yiwenbin@njust.edu.cn

B. Ouyang

The School of Science, Nanjing University of Science and Technology, Nanjing 210094, China


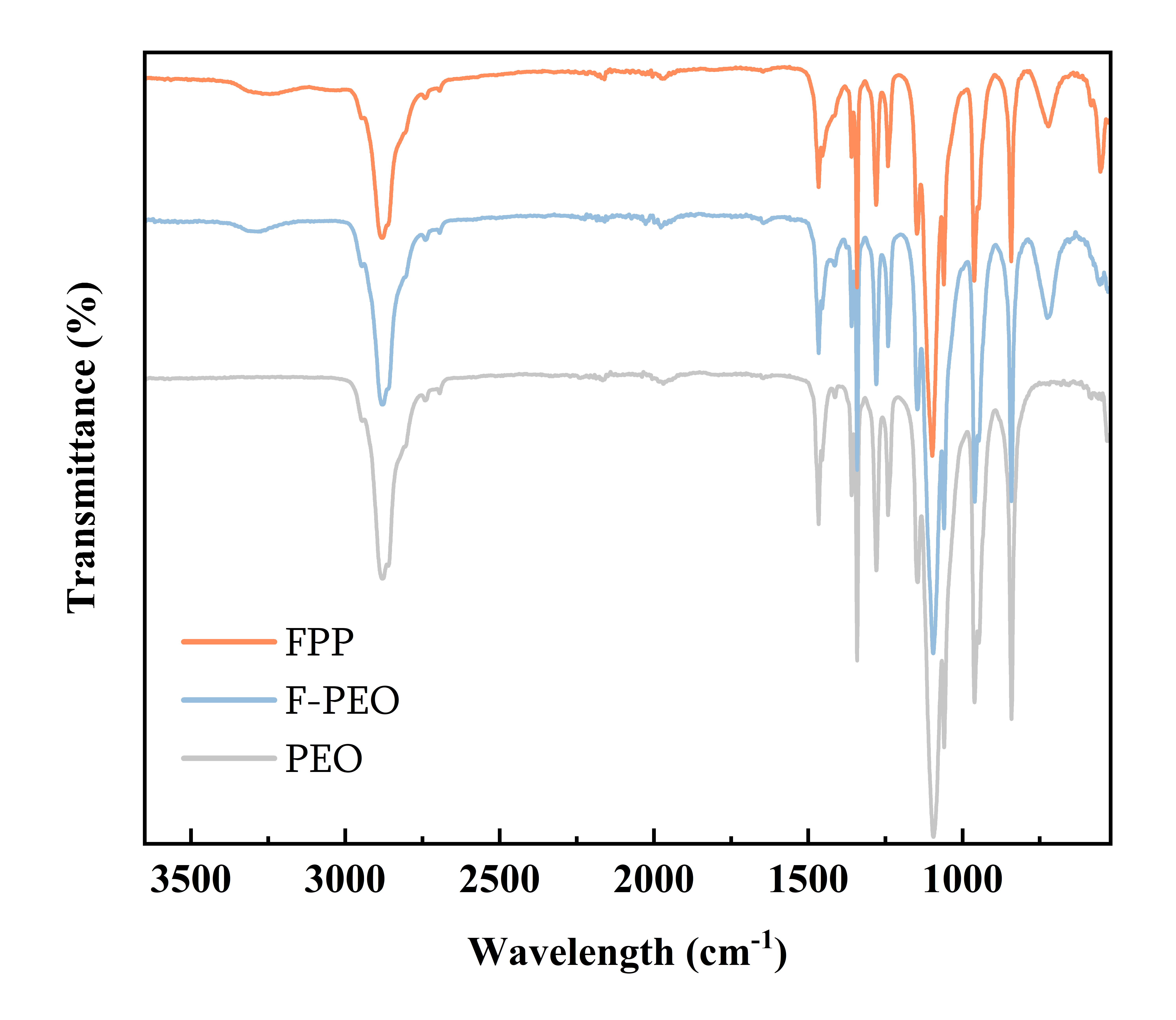


**Figure S1.**

FT-IR spectra of FPP, F-PEO and PEO.


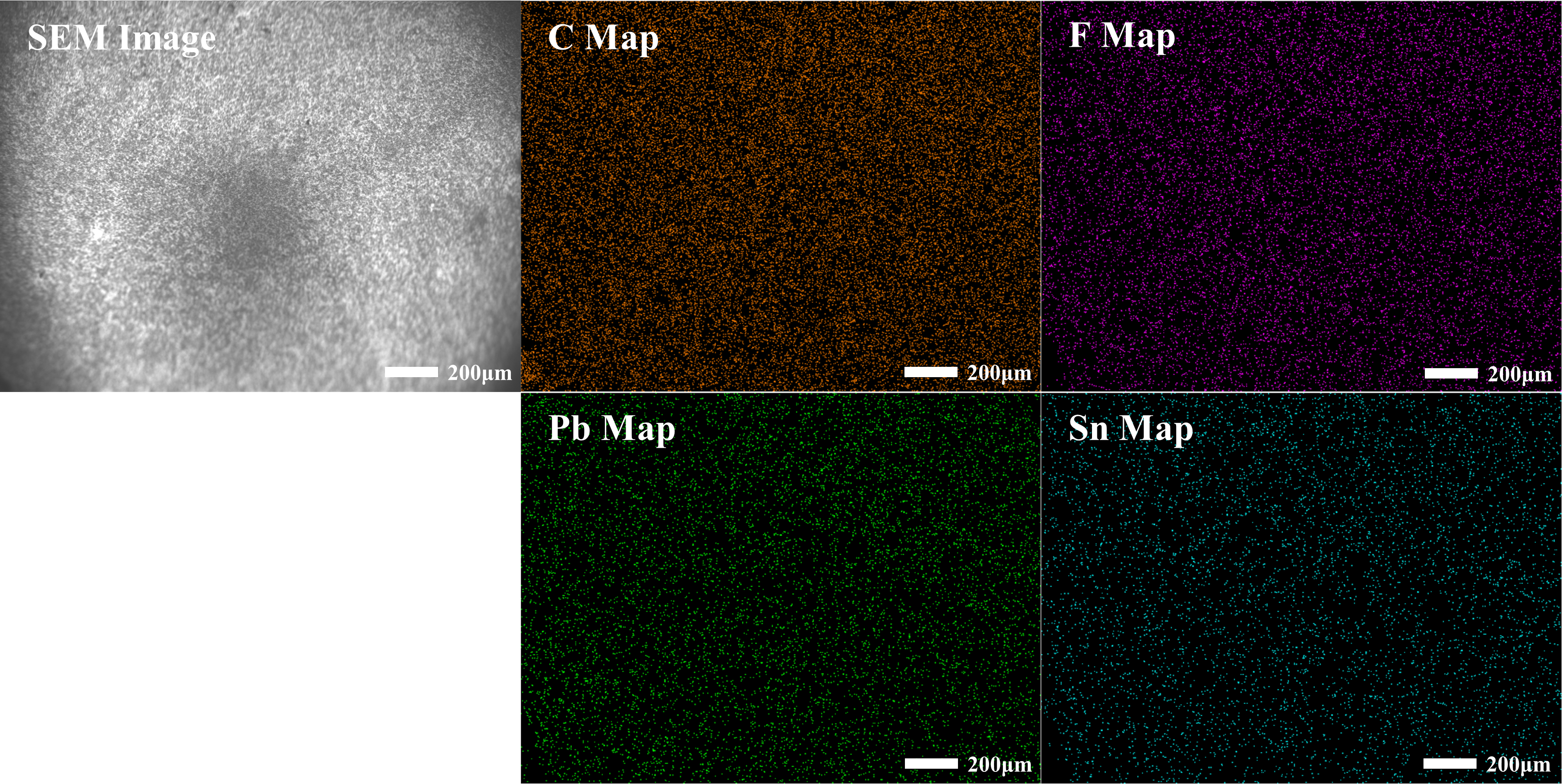


**Figure S**2.

Large-scale SEM and EDS mappings of FPP composite electrolyte.


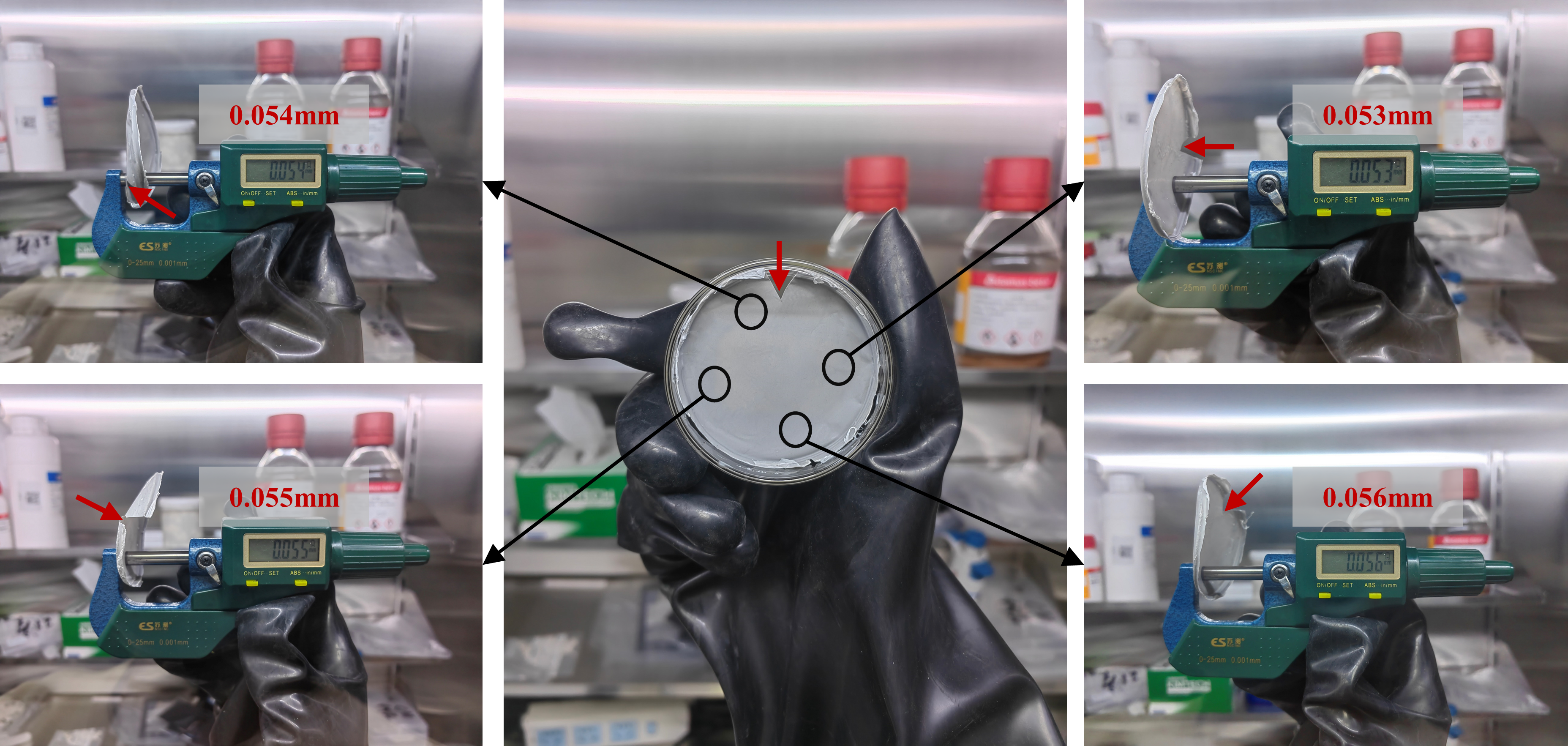


**Figure S**3.

Images of FPP electrolytes prepared via casting and the thickness (use red arrows to highlight the triangular notch used as a marker, measured at four separate locations).


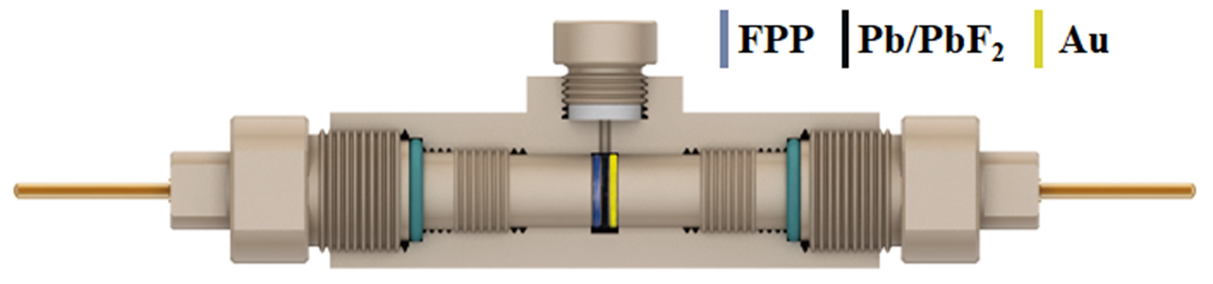


**Figure S**4.

Schematic of Swagelok cell construction (GC|FPP|Pb/PbF_2_|Au|GC) for LSV testing.


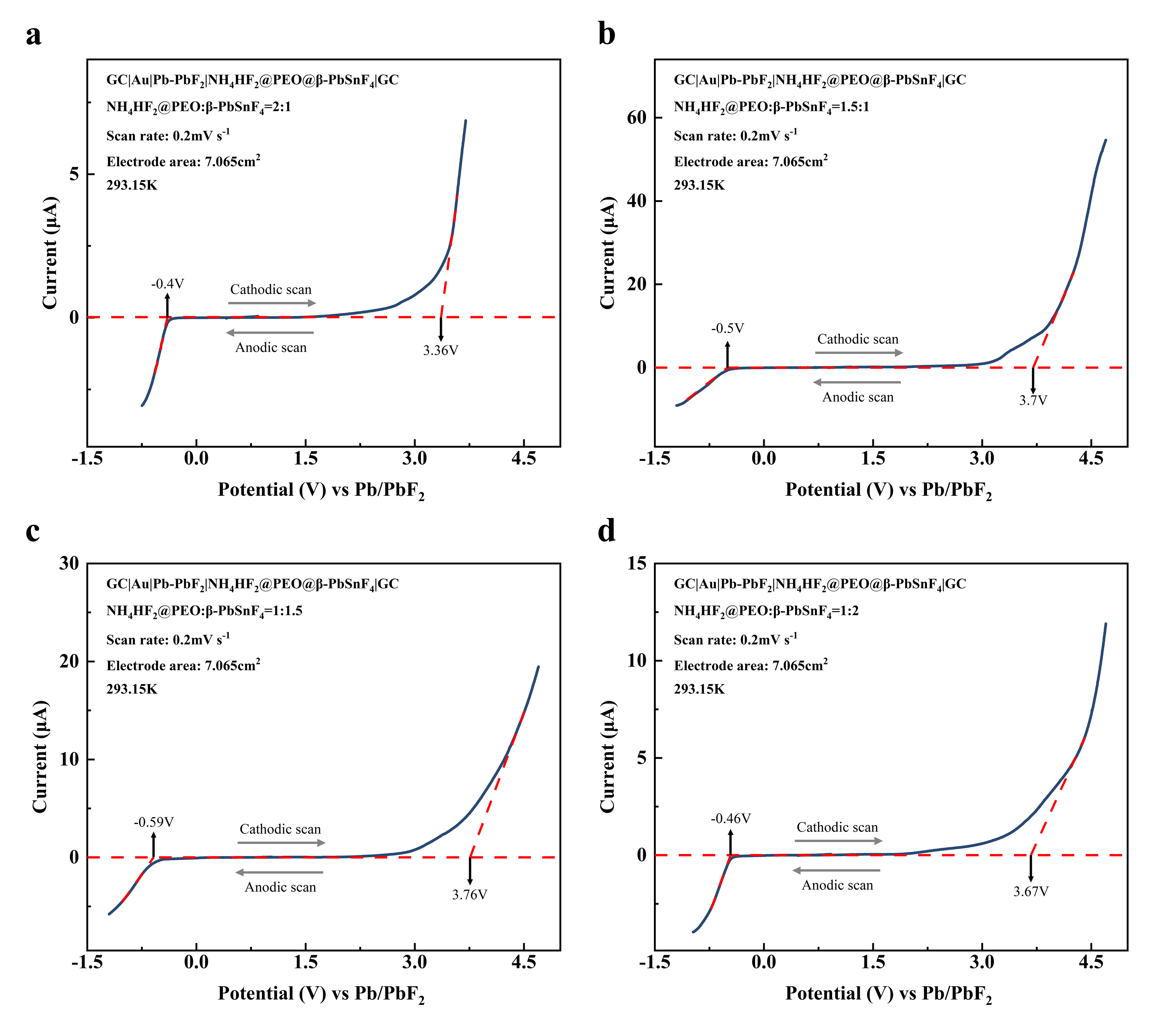


**Figure S5.**

Electrochemical stability (vs Pb/PbF_2_) of FPP composite electrolytes with varying mass ratios. a) 2:1. b)1.5:1. c)1:1.5. d)1:2.

**Table S1.**

Summary of the electrochemical stability (vs Pb/PbF_2_) of FPP composite electrolytes with varying mass ratios (F-PEO: β-PbSnF_4_).

| Mass Ratio | Oxidation Resistance (V) | Reduction Resistance (V) | ESW  (V) | Figure number |
| --- | --- | --- | --- | --- |
| 2:1 | -0.40 | 3.36 | 3.76 | Figure S5a |
| 1.5:1 | -0.50 | 3.70 | 4.20 | Figure S5b |
| 1:1 | -0.73 | 3.86 | 4.59 | Figure 3a |
| 1:1.5 | -0.59 | 3.76 | 4.35 | Figure S5c |
| 1:2 | -0.46 | 3.67 | 4.14 | Figure S5d |


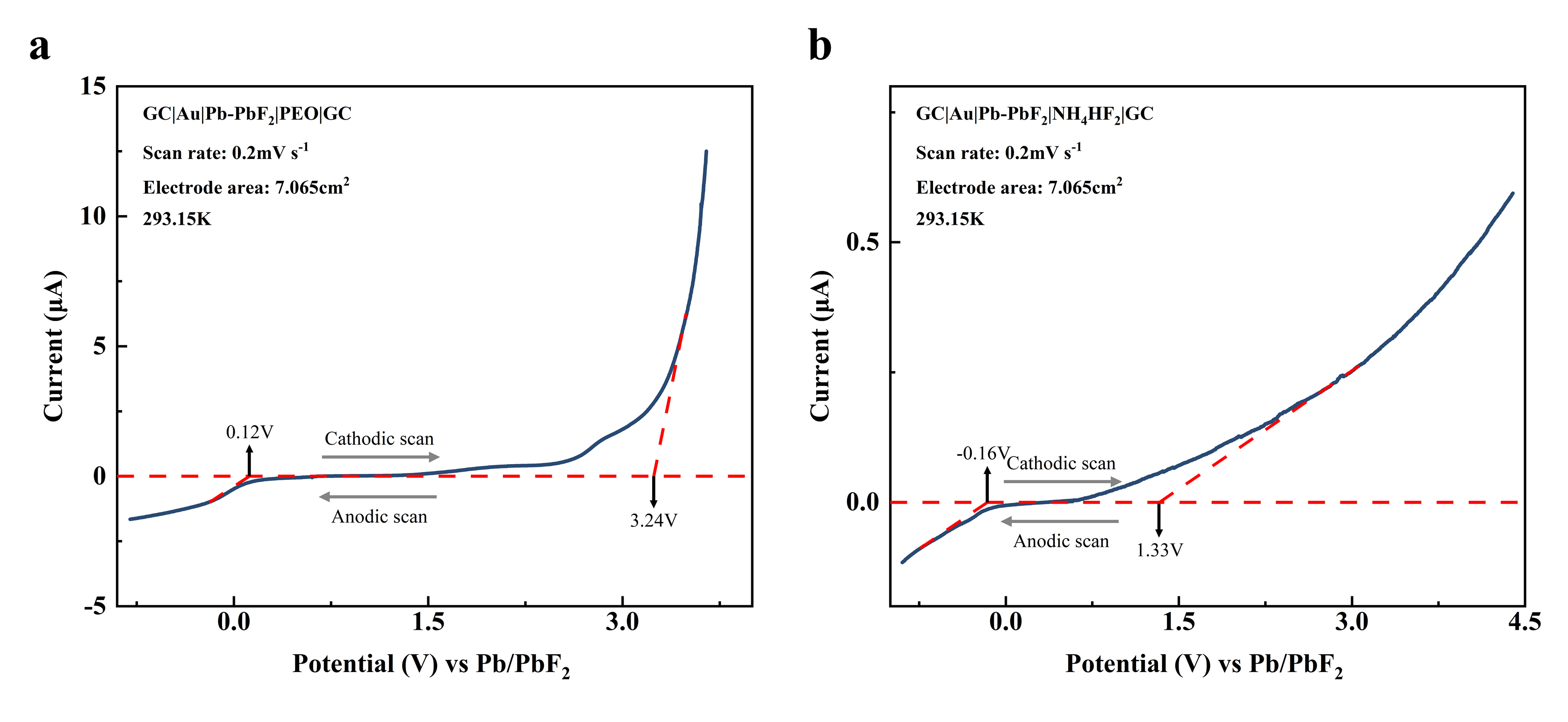


**Figure S6.**

Electrochemical stability (vs Pb/PbF_2_) of single component in FPP composite electrolyte. a) PEO. b) NH_4_HF_2_.

**
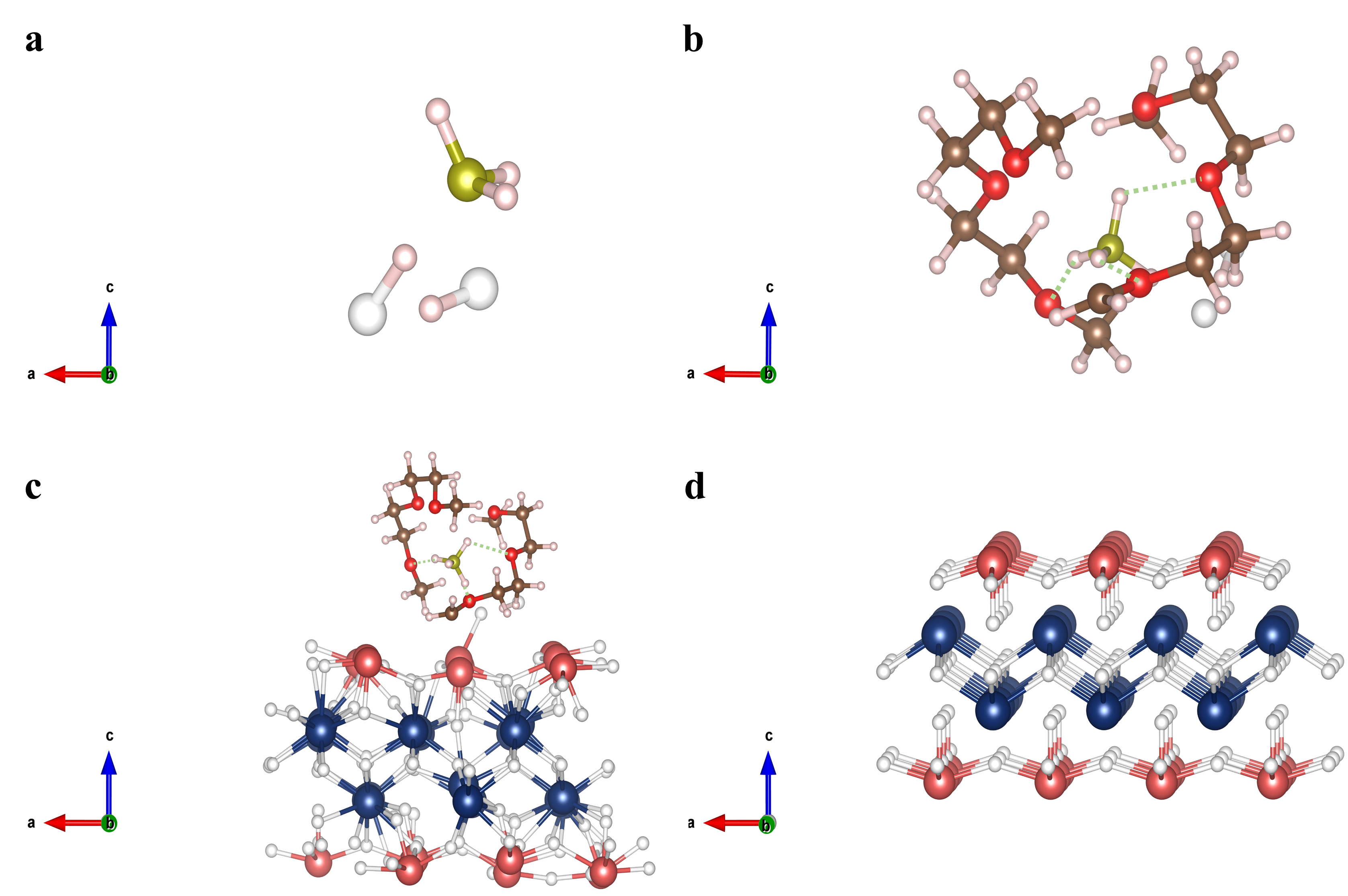
**

**Figure S7.**

The structural models used in the calculations. a) NH_4_HF_2_. b) F-PEO. c) FPP. d) β-PbSnF_4_-surface.


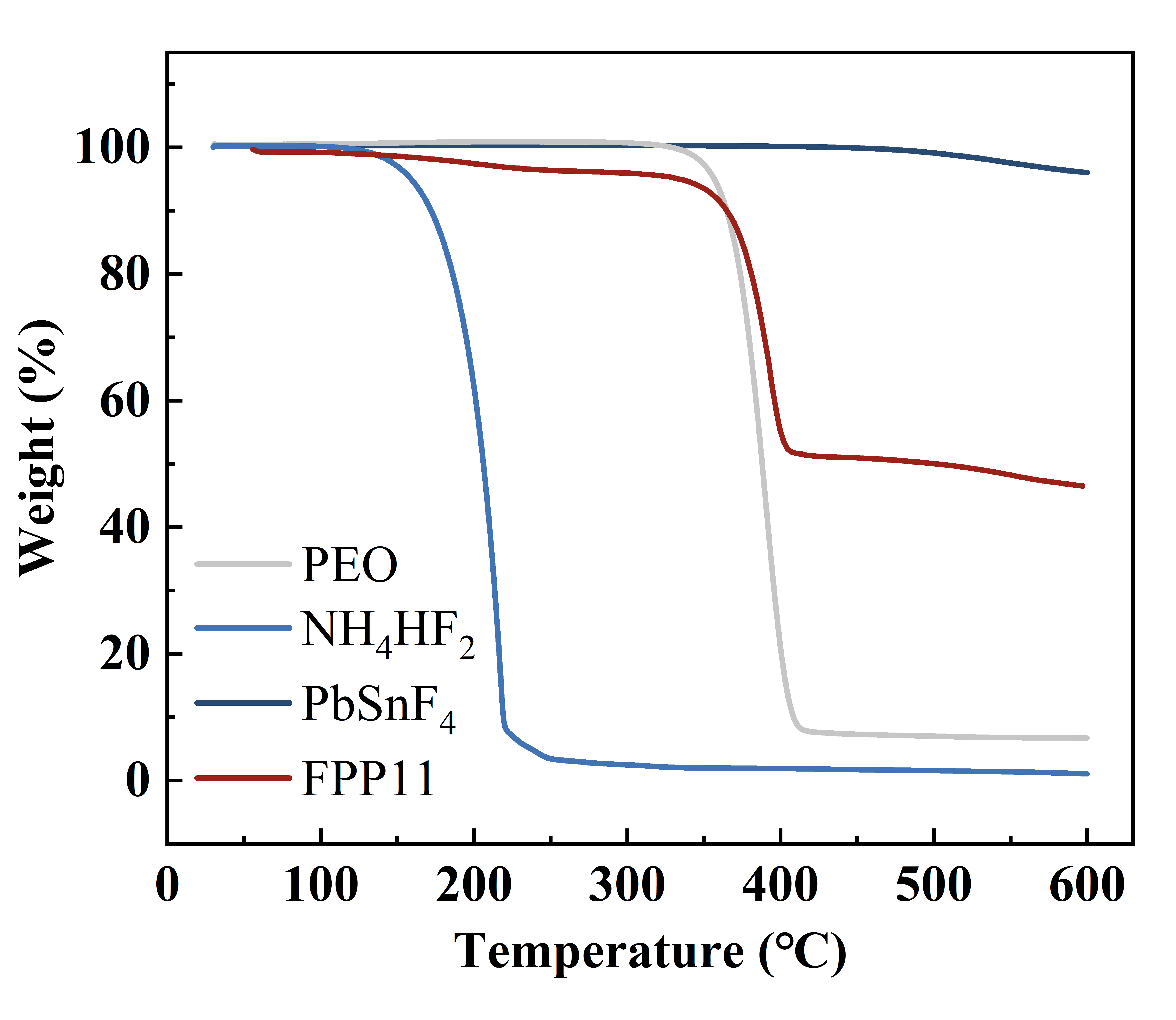


**Figure S8.**

Thermal stability of FPP11 and its raw materials


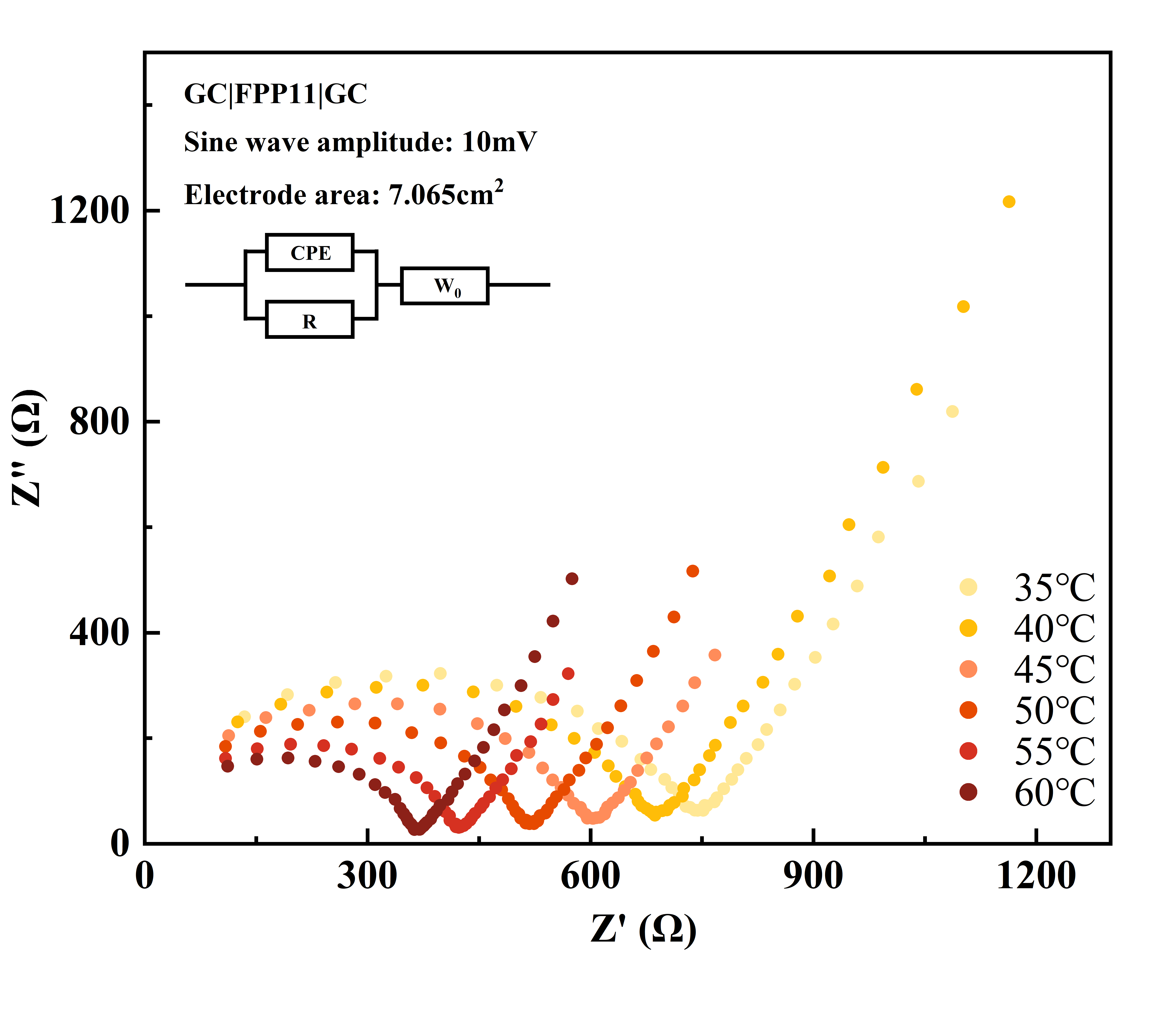


**Figure S9.**

Nyquist plots of FPP11 composite electrolyte at elevated temperatures (VTF behaviors).


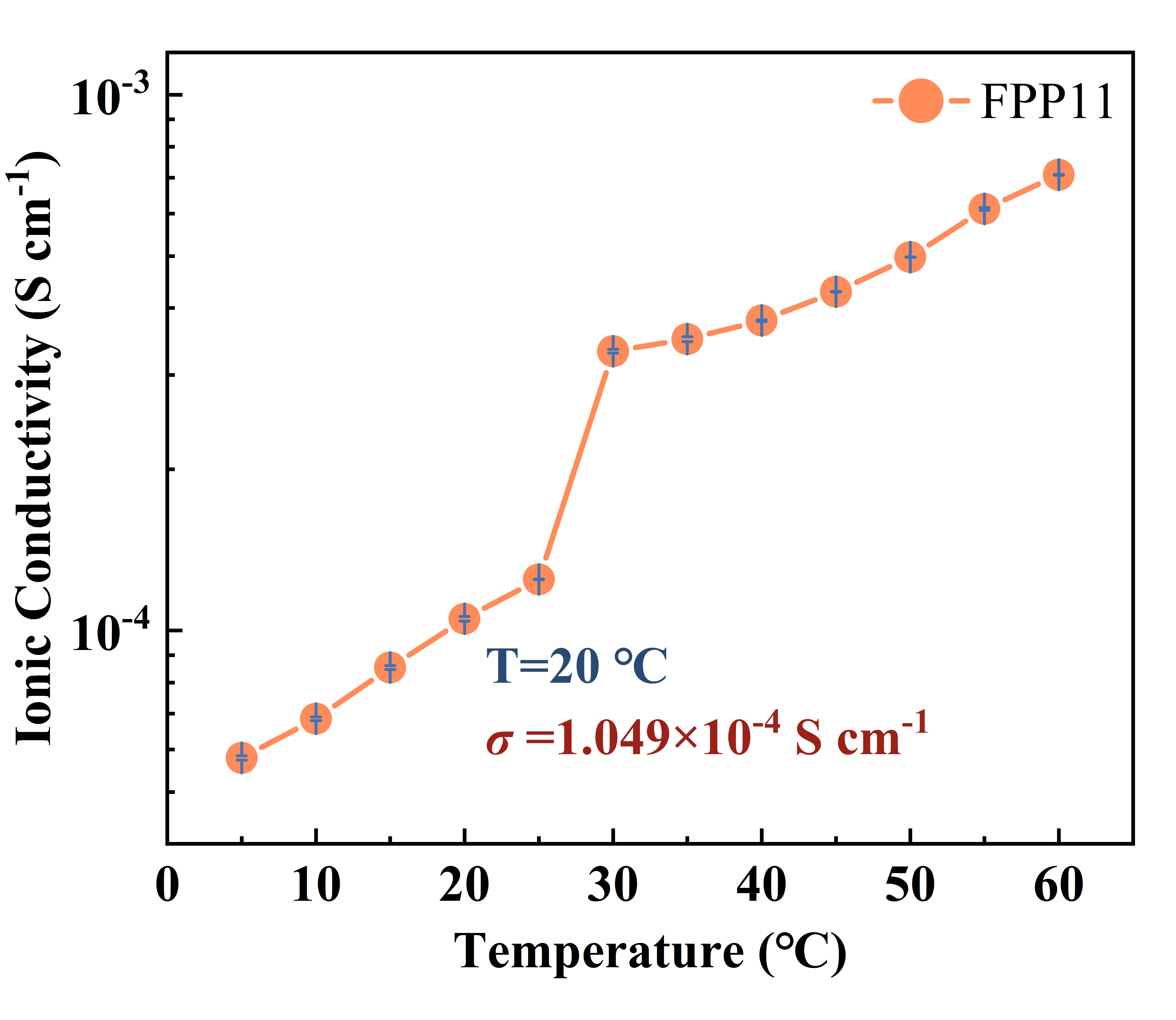


**Figure S10.**

Alternating-current conductivity as a function of temperature.


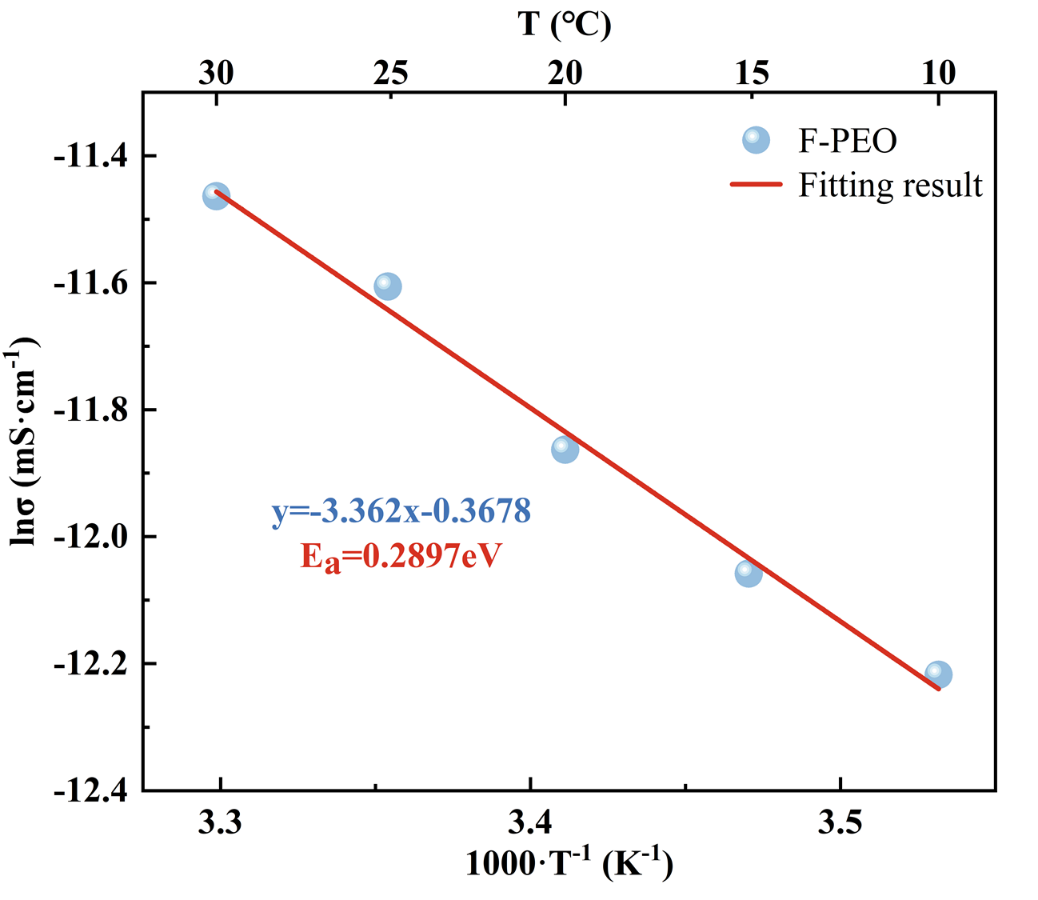


**Figure S11.**

The Arrhenius plot of F-PEO electrolyte used to estiamte activation energy.


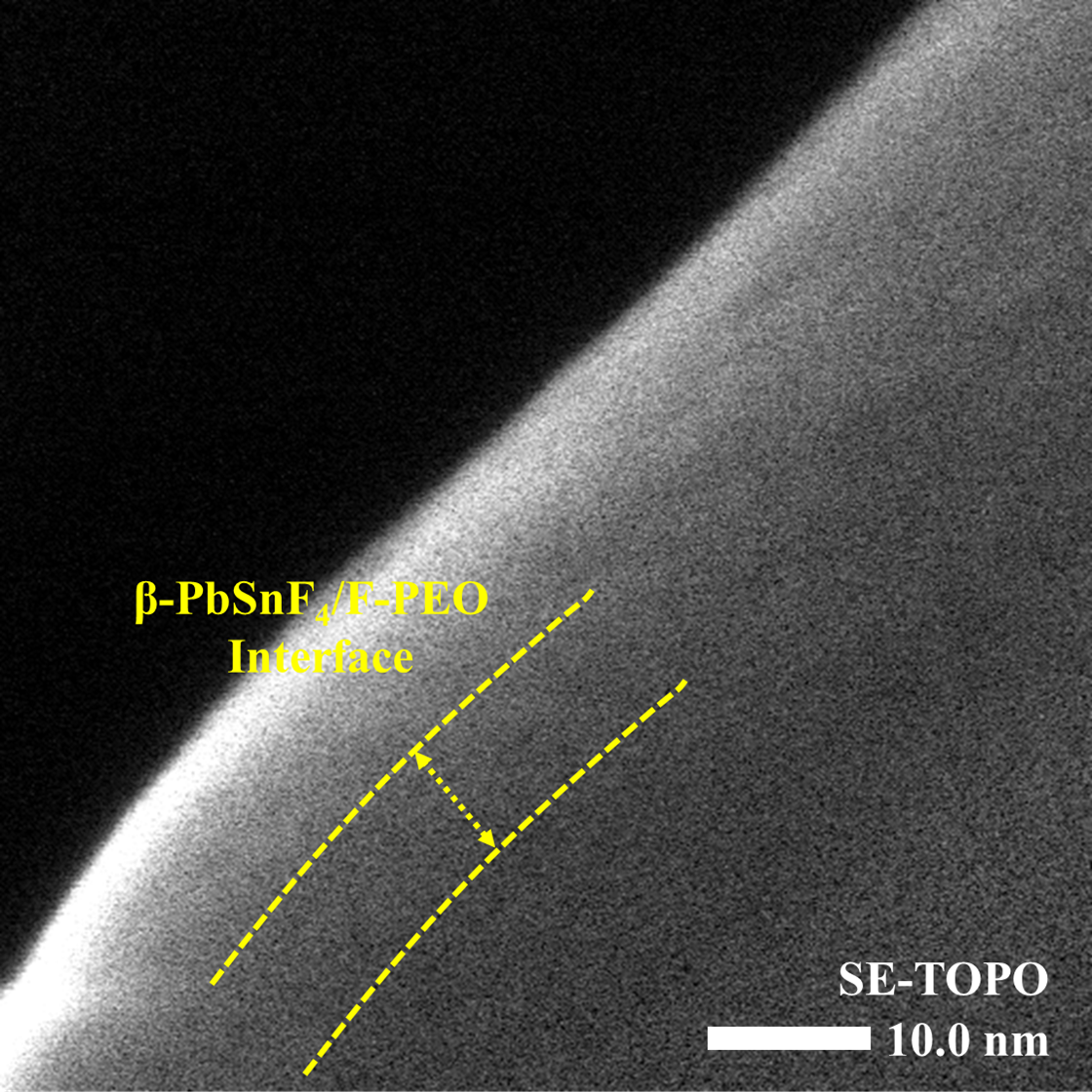


**Figure S12.**

The secondary electron image of the same region as Figure 4e (retains the same interface layer labeling for ease of reference).


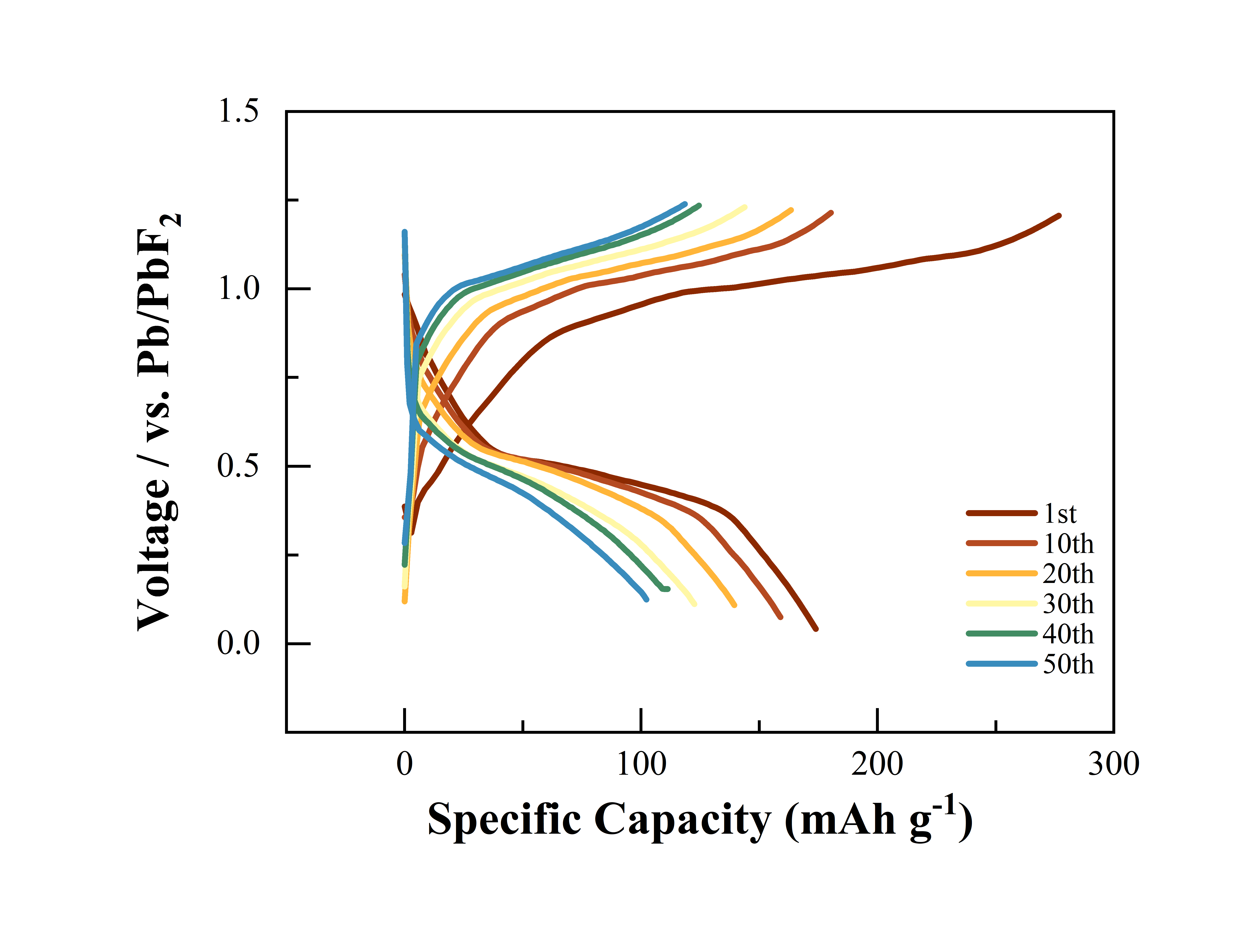


**Figure S13.**

Charge and discharge curves (50 mA g^-1^) of Cathode|FPP11|Anode coin cell at RT.

**Table S2.**

Comparison of this work with the conductivity and electrochemical stability of representative ceramic electrolytes in the near-room-temperature zone.

| **Electrolyte** | **Author** | **Conductivity (S cm^-1^)** | **ESW (V)** |
| --- | --- | --- | --- |
| La_0.9_BaF_2.9_ | Fichtner et al.^[1]^ | 1.0 × 10^−6^ | 2.5 vs BiF_3_ |
| Ba_0.98_Nd_0.02_SnF_4.02_ | Liu et al.^[2]^ | 5.8 × 10^-4^ | 1.5 vs BiF_3_ |
| Ba_0.98_Eu_0.02_SnF_4.02_ | Zang et al.^[3]^ | 3.8 × 10^-4^ | 1.5 vs BiF_3_ |
| BaSnF_4_ | Mohammad et al.^[4]^ | 3.5 × 10^-4^ | 0.45 vs Pb/PbF_2_ |
| γ-PbSnF_4_ | Fujisaki et al.^[5]^ | 3.4 × 10^-4^ | 0.53 vs Pb/PbF_2_ |
| β-PbSnF_4_ | Liu et al.^[6]^ | 1.5 × 10^-3^ | 1.2 vs Pb/PbF_2_ |
| CsPb_0.9_K_0.1_F_2.9_ | Wang et al.^[7]^ | 1.2 × 10^-3^ | 1.8 vs Pb/PbF_2_ |
| Ba_0.96_Mn_0.04_SnF_4_ | Zang et al.^[8]^ | 2.7 × 10^-4^ | 0.13 vs BiF_3_ |
| FPP11 | this work | 1.049 × 10^-4^ | 4.59 vs Pb/PbF_2_ |


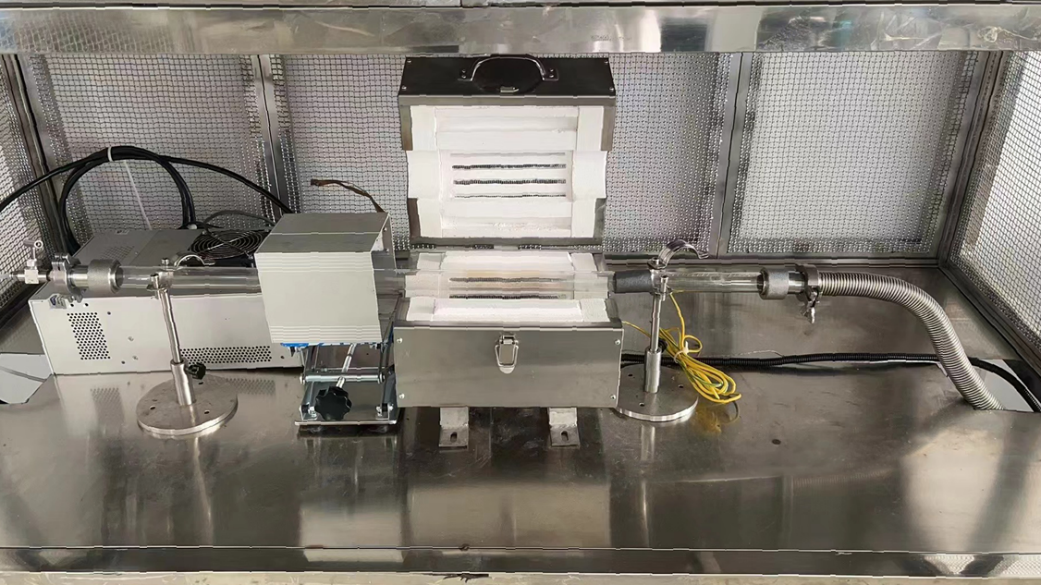


**Figure S14.**

Experiment scene of plasma equipment working area.


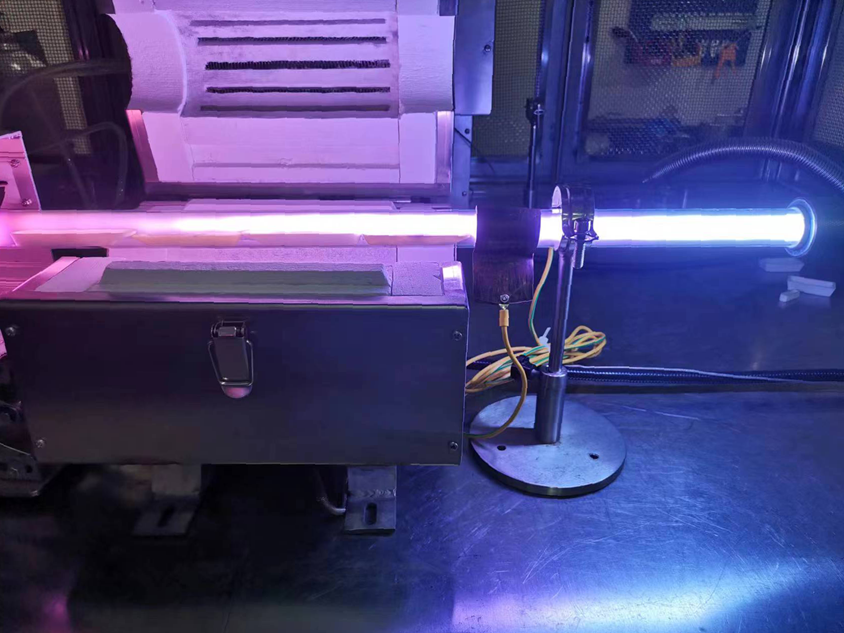


**Figure S15.**

Photographs of plasma equipment in working condition.


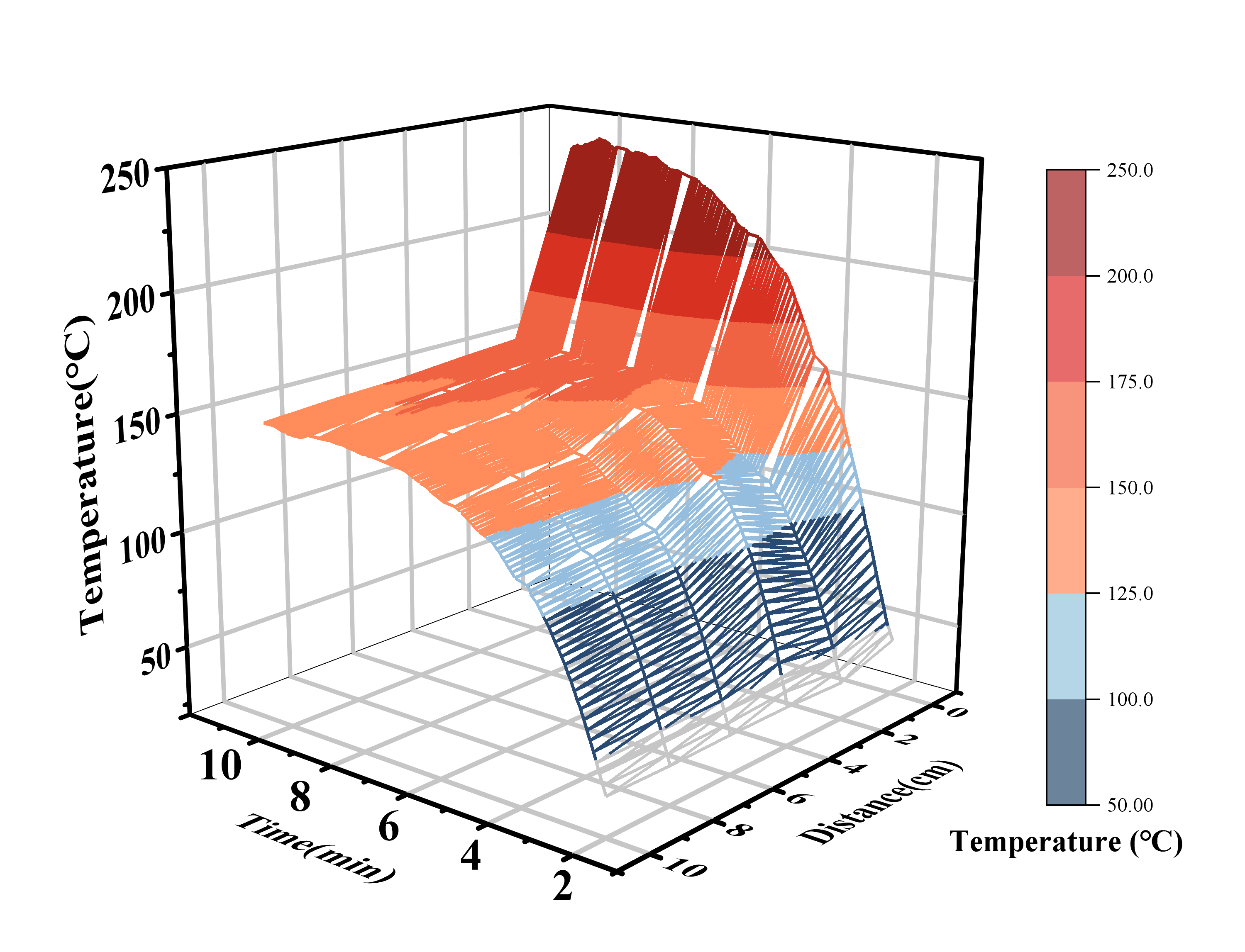


**Figure S16.**

Plasma temperature variation over time/distance from the anode under the conditions in this work.

References

[1] M. A. Reddy, M. Fichtner, *J. Mater. Chem.* **2011**, 21, 17059.

[2] L. Liu, L. Yang, D. S. Shao, K. L. Luo, C. F. Zou, Z. G. Luo, X. Y. Wang, *Ceram. Int.* **2020**, 46, 20521.

[3] Z. H. Zang, L. Liu, L. Yang, K. L. Luo, C. F. Zou, X. Y. Chen, X. Y. Tao, Z. G. Luo, B. B. Chang, X. Y. Wang, *ACS Sustainable Chem. Eng.* **2021**, 9, 12978.

[4] I. Mohammad, R. Witter, M. Fichtner, M. Anji Reddy, *ACS Appl. Energy Mater.* **2018**, 1, 4766.

[5] F. Fujisaki, K. Mori, M. Yonemura, Y. Ishikawa, T. Kamiyama, T. Otomo, E. Matsubara, T. Fukunaga, *J. Solid State Chem.* **2017**, 253, 287.

[6] J. Liu, L. Yi, X. Chen, Y. Tang, Z. Zang, C. Zou, P. Zeng, D. Li, J. Xia, S. Ni, X. Wang, *ACS Appl. Mater. Interfaces* **2023**, 15, 36373.

[7] J. Wang, J. Hao, C. Duan, X. Wang, K. Wang, C. Ma, *Small* **2022**, 18, e2104508.

[8] Z. Zang, J. Liu, X. Tao, C. Zou, X. Chen, L. Yi, B. Chang, X. Wang, *J. Electroanal. Chem.* **2023**, 930.
